# Mapping the landscape of implementation science training: gaps and opportunities to strengthen capacity for implementation research and practice

**DOI:** 10.3389/frhs.2026.1787246

**Published:** 2026-09-22

**Authors:** Hannah Begna, Helene Chokron Garneau, Sobia Khan, Mark McGovern, Julia Moore

**Affiliations:** 1 Stanford Center for Dissemination and Implementation, Stanford University School of Medicine, Palo Alto, CA, United States; 2 The Center for Implementation, Toronto, ON, Canada

**Keywords:** capacity building, evidence-based practice, implementation practice, implementation science, training programs

## Abstract

**Introduction:**

To (1) summarize the current landscape of implementation science (IS) training in North America, (2) describe participant characteristics from The Center for Implementation (TCI), one of the largest IS training programs, and (3) identify opportunities to inform future capacity-building efforts.

**Methods:**

We conducted a structured environmental scan of North American IS training programs; online surveys were then distributed to all identified programs to collect information on program characteristics and target audience. We then performed descriptive and qualitative analyses of the survey results and de-identified demographic and participant response data from TCI course participants.

**Results:**

Fifty-seven IS training programs were identified across North America. Most programs (59.6%) were located in the United States and were primarily graduate level (54.4%), certificate programs (36.8%), and fellowships or post-doctoral opportunities (29.8% each). Nearly half (47.4%) enrolled ≤10 participants per cohort, and approximately 10% enrolled more than 50 participants. Nearly all programs admitted academic audiences (94.7%), with fewer targeting participants from public health (57.9%) or healthcare providers (43.9%). TCI participants (*N* = 1,632 for demographic data; *N* = 436 for qualitative analysis) were a diverse audience representing multiple sectors, most frequently healthcare (26.9%), IS (17.6%), public health (17.0%), and education (15.6%). Organizational affiliations included nonprofit organizations (22.7%), healthcare institutions (18.1%), higher education (17.7%), and government (15.9%). Through qualitative analysis of TCI participant introductions, we identified four major themes reflecting participants' professional contexts and motivations for IS training: (1) system roles, (2) current implementation activities, (3) learning motivations, and (4) application of IS concepts to their work.

**Discussion:**

Current IS trainings in North America are primarily focused on a research audience. Expanding IS implementation training and education to a broader audience, in particular implementation support practitioners who are not engaged in research, but who seek to apply IS to solve problems, is warranted.

## 1 Introduction

Over the past two decades, implementation science (IS) has advanced substantially. Supported by a growing body of theoretical frameworks, methodologies, and studies, IS aims to improve the effectiveness, pace, and sustainability of implementation efforts (1–3). With this progress has come a rising interest in the field, reflected in increased investment in IS research by funding agencies and the development of training infrastructure within academic institutions (2, 4). Yet, translating research into equitable and sustained practice remains challenging. This underscores the need for capacity-building among researchers and professionals.

Capacity building in IS seeks to strengthen the knowledge and skills needed to design and apply the most rigorous methods of IS in research and also implementation practice (5, 6). This can occur in various educational and training structures such as degree and non-degree programs at academic institutions, certificate programs, training institutes, and workshops or conferences (1, 5, 7). Historically, most IS capacity-building efforts have been geared towards academic audiences (i.e., researchers, faculty, and trainees), such as the Implementation Research Institute (IRI), The Society for Implementation Research Collaboration, and The Dissemination and Implementation Science Training Institute (8–11). These individuals desire to apply implementation theories, models, frameworks, and methods in controlled research projects (4, 8, 9, 12). However, the true benefits of IS are relevant for a much broader range of actors, including health system leaders, policymakers, clinicians, community organizations, those supporting implementation (e.g., through intermediaries or within their organizations), and implementation support practitioners (13–15). It remains unclear whether the current landscape of IS training adequately serves the diverse professionals responsible for implementing evidence in practice. This distinction matters because the competencies required for implementation research aren't always the same as those needed to execute implementation (13, 16). Implementation researchers aim to generate and test theories, models, and methods; implementation support practitioners apply these approaches in real-world settings. As a result, a knowledge-practice gap may be emerging. The field designed to close the evidence-practice gap may itself be producing knowledge that practitioners cannot readily access or apply (17). This creates a broader tension for capacity-building initiatives, which must be responsive to the needs of both groups, as well as individuals who occupy hybrid roles spanning research and practice.

Previous reviews have documented capacity-building programs in IS. However, little is known about how programs differ in target audience (including those outside of academia or research), scope, and structure. These factors may ultimately shape who has access to formal training and how effectively participants can apply the knowledge and skills they acquire (4). Thus, a comprehensive and updated review of existing IS training opportunities is needed to describe what is currently offered, identify areas for improvement, and align future capacity-building strategies to diverse professional settings.

Among capacity-building programs, *The Center for Implementation* (TCI) is one of the largest providers of IS training (18), reaching a global audience of both researchers and implementation support practitioners through online courses and a certificate program. To date, there is limited understanding of non-academic capacity-building efforts such as TCI, though their participant sample is often similar or larger than academic trainings. As one of the largest training programs in North America, TCI serves as a compelling case example for examining characteristics and needs of a cross-sector implementation support workforce. To contribute to the knowledge base of IS capacity-building programs, we aim to (1) summarize the current landscape of training programs in North America and their key characteristics; (2) describe TCI's participant audience; and, (3) identify opportunities to inform future capacity-building efforts, particularly for audiences beyond academia who remain underrepresented but essential to advancing the impact of IS.

## 2 Methods

This study employed a three-part approach: (1) a structured environmental scan of current IS training programs in North America, (2) a survey administered to identified programs, and (3) descriptive and qualitative analyses of internal participant data from The Center for Implementation.

### 2.1 Identification of IS training programs

A structured environmental scan was conducted to identify all current IS training programs in North America. Searches were carried out between May and September 2025 using multiple sources including Stanford University Library databases, Google, Google Scholar, and institutional websites.

Search terms combined core IS terminology with synonymous or related concepts. Primary terms included “*Implementation Science/Practice*” and “*Dissemination and Implementation*” paired with descriptors including *program*, *course*, *certificate*, *training*, *degree*, and *workshop*. Degree variations (e.g., *undergraduate, master's*, *PhD*, *graduate, certificate*) and regional or state-specific modifiers were also tested. Supplementary terms (e.g., *webinar*, *training program*, *modules*, *seminar*, *implementation practice*, *tools*) were used to ensure broad capture. Search results were screened for relevance to IS education, training, and capacity building. Inclusion criteria were: located in North America, explicit IS focus, and recurring training opportunities. Exclusion criteria were: non-English and employer-specific programs, one-time events, and consultation or mentorship only opportunities. A total of *N* = 54 programs were initially identified. Each website was reviewed independently by two research team members to confirm eligibility and identify duplicates. Any discrepancies were resolved through discussion and consensus before finalizing inclusion.

### 2.2 Survey of identified programs

Following initial verification, an online survey was distributed via email to the primary contacts for all eligible programs. The survey collected structured information on: program country and institutional affiliation, training type and level, admission frequency, duration, cohort size, cost, intended audience, course content, and topic specificity. Course content categories were developed *a priori* through a structured synthesis of existing IS competency frameworks (15, 19–23), yielding eleven major topics spanning both implementation research and implementation support practice. Programs were asked to indicate which topics their training addressed.

Responses were reviewed for completeness and accuracy. Four discontinued programs were removed and seven new programs identified through follow-up survey responses were added, resulting in a final sample of *N* = 57 programs.

### 2.3 Quantitative analysis

Descriptive analyses were conducted to summarize program- and participant-level characteristics. Program-level variables were summarized as frequencies and percentages. Internal participant data provided by TCI were analyzed to describe participant characteristics overall and across five courses. Variables included demographic information, professional sector, organizational affiliation, geographic location, and years of experience.

### 2.4 Qualitative analysis

To further examine participant engagement in IS training, a rapid qualitative analysis was conducted on de-identified course introductions submitted by TCI participants in the most popular course during the introductory module. Participant introductions were an optional course activity, and therefore not all StrategEase participants submitted an introduction. The qualitative analysis included the subset of participants who provided an introduction. Consistent with established rapid approaches, this method was selected to generate timely insights from participant data while maintaining methodological rigor (24, 25).

We first developed a summary template to guide data synthesis and ensure systematic documentation. Two research team members independently reviewed a sample of introductions to develop an initial codebook grounded in pre-defined content themes. A combined inductive and deductive strategy was used as consistent with existing rapid qualitative methods (24, 25). Two members of the research team independently coded the data. Coding discrepancies were resolved through discussion and consensus until agreement was reached. Through iterative discussion, code definitions were refined to ensure consistency and reliability. The finalized codebook was systematically applied to the full dataset. Coders compiled structured summaries after independent review and calibrated their approaches through jointly reviewing and comparing previous versions to enhance rigor and dependability (24, 25). Emergent themes were then analyzed across participants, using matrices organized around key domains from the summary template. Representative quotes were selected to illustrate each theme.

### 2.5 Quality assurance and ethical considerations

Throughout all phases, multiple steps were taken to enhance rigor and consistency. Screening and coding procedures were double-checked by independent reviewers with discrepancies resolved through consensus meetings. Survey data were cross validated against publicly available program information. Qualitative coding reliability was verified through periodic discussions, and all analytic decisions were documented to ensure reproducibility.

This study was determined to be exempt by Stanford University's institutional review board, as it involved the analysis of publicly available program information and de-identified internal data from The Center for Implementation. Although de-identified program data were provided directly by The Center for Implementation, all analyses were conducted independently by the research team to reduce potential bias and strengthen internal validity. No personally identifiable information was collected or analyzed.

## 3 Results

### 3.1 Environmental scan and survey

A total of 57 IS training programs were identified and surveyed across North America (Table 1). The majority of North American programs (59.6%) were located in the United States and delivered in-person only; 35.1% of programs were offered as hybrid or remote. Most were offered at the graduate level (54.4%), followed by professional development workshops (42.1%), certificate programs (36.8%), and fellowships or post-doctoral opportunities (29.8% each). Nearly half (47.4%) of trainings admitted small cohorts of ≤10 participants per cycle, with approximately 10% enrolling more than 50 participants. While most training opportunities were $0 (42.1%)—no cost to participants, due to external funding from federal agencies such as the National Institutes of Health—over one third had tuition costs exceeding $7,000 USD (35.1%). Among programs with tuition, mean cost was $23,650 ($31,598); median cost was $8,900. Regarding the intended audience, nearly all programs (94.7%) offered admission to those in academia (i.e., faculty, researchers, students, and postdoctoral scholars). Several programs also offered or targeted training to those in public health (57.9%) and healthcare providers (43.9%); fewer programs welcomed other audiences from the education sector (24%), government (24%), and policy (26%). Training content covered a wide range of IS topics. Nearly all programs addressed implementation strategies (96.5%), frameworks and theories (94.7%), and foundations of IS (93.1%). Fewer programs incorporated capacity building and team composition (50.9%), economic or policy considerations (47.4%), or de-implementation (40.4%). Approximately one-third of training (31.6%) were topic- or sector-specific, while most remained general in focus (68.4%). Additional detail from survey responses can be found in Supplementary Table S1.

**Table 1:** Characteristics of current implementation science trainings in North America.

Characteristic	Category	*N* (Total = 57)	%
Country	Canada	3	5.3
	Flexible/Hybrid	10	17.5
	Remote (online)	10	17.5
	United States	34	59.6
Training level	Certificate program	21	36.8
	Fellowship	17	29.8
	Graduate level	31	54.4
	Post-doctoral	17	29.8
	Professional development workshop/training	24	42.1
	Undergraduate level	6	10.5
	Other (webinar series, reading course, etc.)	5	8.8
Admission frequency	Annually	38	66.7
	Biannually (twice per year)	4	7.0
	Monthly	1	1.8
	Multiple offerings per year	4	7.0
	Rolling enrollment	10	17.5
Cost	Funded	24	42.1
	<$3,000	8	14.0
	$3,000—$7,000	5	8.8
	$7,001—$25,000	11	19.3
	>$25,000	9	15.8
Cohort size	1–10	27	47.4
	11–20	15	26.3
	21–50	9	15.8
	51–100	4	7.0
	100+	2	3.5
Duration	1–7 days	12	21.1
	A few months	4	7.0
	Six months—1 year	10	17.5
	More than 1 year	30	52.6
	Self-paced	1	1.8
Audience	Academia (faculty, researchers, students, postdocs)	54	94.7
	Community organizations	20	35.1
	Education sector professionals	14	24.6
	Funding agencies/Government institutions	14	24.6
	Healthcare institutions	17	29.8
	Healthcare providers	25	43.9
	Human services professionals	20	35.1
	Policy makers	15	26.3
	Public health professionals	33	57.9
	Other	6	10.5
Competencies	Adaptation and Fidelity	44	77.2
	Assessing Context and Readiness	48	84.2
	Capacity Building and Team Composition	29	50.9
	De-implementation	23	40.4
	Economic and Policy Considerations	27	47.4
	Foundations of Implementation Science	53	93.0
	Frameworks and Theories	54	94.7
	Implementation Strategies	55	96.5
	Measurement and Outcomes	50	87.7
	Stakeholder and Community Engagement	52	91.2
	Study Design and Methods	49	86.0
	Other	5	8.8
Topic specificity	No (generalist/agnostic)	39	68.4
	Yes (topic/sector specific)	18	31.6

### 3.2 TCI participant data

Participant data from TCI revealed a diverse and international audience (Table 2). Participants represented a broad range of age groups, mostly aged 35–44 years (36.2%) and 45–54 years (27.3%). Across all courses, 85.6% of participants identified as women and 67.1% as White. Participants most commonly worked in healthcare (26.9%), IS (17.6%), public health (17%), and education (15.6%), with many reporting multiple disciplinary affiliations (56.4%). Representation included diverse organizations, including nonprofit organizations (22.7%), healthcare institutions (18.1%), higher education (17.7%), government agencies (15.9%), and research organizations (17.4%), with substantial overlap across sectors (36.6%). Professional experience varied widely, spanning early- to late-career stages (1–5 years: 28.1%; 6–10 years: 19.8%; 11–15 years: 18.8%; 16+ years: 28.1%).

**Table 2:** Participant characteristics of the center for implementation's implementation science certificate program, overall and by course.

*N* (%)	All courses (*N* = 1,632)	StrategEase: The HOW of creating sustainable change (*N* = 652)	Implementation, spread, and scale (*N* = 324)	Cultivating trust and navigating power (*N* = 275)	Embracing complexity (*N* = 341)	Igniting change from the middle (*N* = 40)
Age group						
18–24	31 (2%)	17 (2.8%)	7 (2.3%)	3 (1.1%)	2 (0.6%)	2 (5.6%)
25–34	329 (21.3%)	146 (24%)	73 (23.5%)	34 (12.7%)	63 (19.5%)	13 (36.1%)
35–44	560 (36.2%)	233 (38.3%)	109 (35.2%)	110 (41%)	96 (29.7%)	12 (33.3%)
45–54	422 (27.3%)	144 (23.6%)	91 (29.4%)	83 (31%)	98 (30.3%)	6 (16.7%)
55–64	164 (10.6%)	56 (9.2%)	20 (6.5%)	34 (12.7%)	51 (15.8%)	3 (8.3%)
65–75	31 (2%)	9 (1.5%)	5 (1.6%)	4 (1.5%)	13 (4%)	NA
Over 75	1 (0.1%)	1 (0.2%)	NA	NA	NA	NA
Prefer not to disclose	8 (0.5%)	3 (0.5%)	5 (1.6%)	NA	NA	NA
Country						
Australia	134 (8.6%)	63 (10.1%)	19 (6.1%)	19 (7.2%)	30 (9.1%)	3 (7.9%)
Canada	653 (41.8%)	302 (48.4%)	138 (44.7%)	77 (29.3%)	124 (37.7%)	12 (31.6%)
United States	535 (34.2%)	202 (32.4%)	103 (33.3%)	114 (43.3%)	100 (30.4%)	16 (42.1%)
Other	241 (15.4%)	57 (9.1%)	49 (15.9%)	53 (20.2%)	75 (22.8%)	7 (18.4%)
English as First Language	1,149 (77.8%)	457 (79.2%)	230 (76.4%)	193 (77.2%)	244 (77.7%)	25 (71.4%)
Gender						
Man	200 (12.5%)	83 (13.1%)	37 (11.6%)	37 (13.6%)	38 (11.3%)	5 (12.5%)
Non-binary	16 (1.0%)	3 (0.5%)	6 (1.9%)	3 (1.1%)	3 (0.9%)	1 (2.5%)
Woman	1,370 (85.6%)	540 (85.4%)	270 (84.9%)	231 (84.6%)	293 (87.5%)	34 (85.0%)
Prefer not to disclose	14 (0.9%)	6 (1.0%)	5 (1.6%)	2 (0.7%)	1 (0.3%)	0 (0.0%)
Race/Ethnicity						
Asian	163 (10.4%)	66 (10.6%)	37 (11.9%)	19 (7.1%)	38 (11.8%)	3 (7.5%)
Black	141 (9%)	45 (7.2%)	27 (8.7%)	41 (15.3%)	25 (7.7%)	3 (7.5%)
Hispanic or Latinx	86 (5.5%)	24 (3.8%)	21 (6.7%)	21 (7.8%)	13 (4%)	7 (17.5%)
Indigenous, First Nations, or Metis	9 (0.6%)	2 (0.3%)	NA	3 (1.1%)	4 (1.2%)	NA
Middle Eastern	27 (1.7%)	11 (1.8%)	8 (2.6%)	3 (1.1%)	5 (1.5%)	NA
More than one race	46 (2.9%)	20 (3.2%)	5 (1.6%)	7 (2.6%)	12 (3.7%)	2 (5%)
Prefer not to disclose	44 (2.8%)	15 (2.4%)	14 (4.5%)	6 (2.2%)	8 (2.5%)	1 (2.5%)
White	1,051 (67.1%)	441 (70.7%)	200 (64.1%)	168 (62.7%)	218 (67.5%)	24 (60%)
Field						
Education	503 (15.6%)	168 (13.2%)	96 (14.3%)	128 (23.3%)	98 (15.1%)	13 (14.4%)
Healthcare	868 (26.9%)	380 (30%)	180 (26.9%)	116 (21.1%)	176 (27.1%)	16 (17.8%)
Implementation Science	568 (17.6%)	201 (15.9%)	126 (18.8%)	96 (17.5%)	128 (19.7%)	17 (18.9%)
Justice	117 (3.6%)	60 (4.7%)	25 (3.7%)	14 (2.6%)	15 (2.3%)	3 (3.3%)
Mental Health	354 (11%)	153 (12.1%)	75 (11.2%)	56 (10.2%)	60 (9.2%)	10 (11.1%)
Public Health	547 (17%)	199 (15.7%)	109 (16.3%)	102 (18.6%)	121 (18.6%)	16 (17.8%)
Social Services	267 (8.3%)	106 (8.4%)	58 (8.7%)	37 (6.7%)	51 (7.9%)	15 (16.7%)
Prefer not to disclose	2 (0.1%)	1 (0.1%)	1 (0.1%)	NA	NA	NA
More than one (double counted)	892 (56.4%)	346 (54.5%)	197 (61.9%)	148 (54.6%)	176 (55%)	25 (65.8%)
Organization						
Community Organization	144 (6%)	58 (6.2%)	24 (4.9%)	28 (7.1%)	31 (6%)	3 (5.3%)
For Profit Organization	52 (2.2%)	14 (1.5%)	12 (2.5%)	9 (2.3%)	13 (2.5%)	4 (7%)
Government	381 (15.9%)	162 (17.2%)	72 (14.8%)	66 (16.8%)	75 (14.5%)	6 (10.5%)
Healthcare Institution	434 (18.1%)	178 (18.9%)	92 (18.9%)	60 (15.3%)	100 (19.3%)	4 (7%)
Higher Education	425 (17.7%)	152 (16.1%)	93 (19.1%)	75 (19.1%)	95 (18.4%)	10 (17.5%)
Nonprofit Organization	544 (22.7%)	217 (23%)	112 (23%)	100 (25.5%)	102 (19.7%)	13 (22.8%)
Research Organization	416 (17.4%)	162 (17.2%)	82 (16.8%)	54 (13.8%)	101 (19.5%)	17 (29.8%)
More than one (double counted)	573 (36.6%)	225 (36.1%)	116 (36.6%)	89 (33.7%)	129 (39.9%)	14 (35.9%)
Years of experience						
<1 year	82 (5.2%)	43 (6.9%)	15 (4.8%)	10 (3.8%)	13 (3.9%)	1 (2.6%)
1–5 years	439 (28.1%)	184 (29.7%)	103 (33.2%)	63 (23.8%)	73 (22.1%)	16 (41%)
6–10 years	310 (19.8%)	123 (19.8%)	52 (16.8%)	46 (17.4%)	78 (23.6%)	11 (28.2%)
11–15 years	294 (18.8%)	122 (19.7%)	61 (19.7%)	53 (20%)	53 (16.1%)	5 (12.8%)
16+ years	439 (28.1%)	148 (23.9%)	79 (25.5%)	93 (35.1%)	113 (34.2%)	6 (15.4%)

Qualitative analysis of de-identified participant data from TCI's StrategEase course (*N* = 436), the first course in the Level 1 Implementation Support Specialist Certificate Program revealed four major themes: (1) System roles, (2) Current implementation activities, (3) Learning motivations, and (4) Applying IS course content (Table 3). Learners represented a wide range of professional system roles, reflecting the cross-sector and interdisciplinary reach of IS training audiences and their varying implementation-related activities. Participants' learning motivations included deepening foundational knowledge and acquiring practical tools to strengthen implementation and sustainment processes, while others emphasized professional growth or bridging research and practice. Learners also described applying course content to push organizational change, scale programs and services, improve patient-centered care and access, and strengthen implementation capacity.

**Table 3:** System roles, implementation activities, and learning motivations of participants in an implementation science certificate program.

Themes	Sub-themes	Example quotes
System role	Researchers (implementation and topic-specific) Clinicians & healthcare providers Implementation support practitioners Organizational leaders Policy & government Human services and education professionals Evaluators Students & trainees Hybrid roles	“Hi, — from University of —, in —, UK. I am a clinical exercise physiologist and full time researcher with an interest in implementation of evidence-based strength training guidelines/programmes for public health.” (244) “In my role as policy analyst, I provide training and technical assistance…implementing reentry programs…many of these programs are new and complex, which is where implementation science could really help guide the work and ensure that programs are human-centered and can achieve successful outcomes.” (71) “I am currently an evaluator at the—, a non-profit think tank, where I use my research skills mixed with implementation science to evaluate programs, products, and services aimed to reduce the risk for biased policing against Black and Brown communities and to redesign public safety.” (327)
Current implementation activities	Capacity building, training, and facilitation Coordination and partnership development Collaboration and networking Project evaluation Implementation leadership Knowledge translation and dissemination Organizational change Embedded research Implementation research	“My role is to work with researchers, community partners and the government to embed knowledge translation and implementation into a federally funded initiative examining how to better support seniors and promote healthy aging.” (417) “My role includes patient- and healthcare provider oriented research to inform organizational strategy related to pain management, leading development of clinical standards and practice supports and related implementation supports, and education of providers, patients, and healthcare learners.” (283)
Learning motivations	IS foundations Applied implementation Professional identity and career development Equity and community, patient, and end-user centered implementation Bridging research and practice	“I am excited to see how these strategies and processes will equip me and others to become better ‘Implementation Practitioners’ and support our systems on a larger scale.” (61) “Our work involves conducting needs assessments/root cause analyses in schools to determine a pathway for change. Implementation science is necessary to positively affect systems change in schools to assure students with disabilities are afforded the opportunities and access to a free and appropriate education.” (120) “I'm an aspiring implementation scientist that hopes to one day bridge research and practice!” (372)
Applying implementation science course content	Implementation within organizations Scaling programs, practices, and services Patient-centered care and access Organizational and system capacity building Organizational change	“Our organization is expanding its implementation support nationally. This certification will assist us in our efforts to build implementation capacity and identify, contextualize, and improve evidence-based implementation strategies in various settings.” (384) “Part of my role within the—within this initiative is to build capacity and support local government areas to use systems thinking processes to engage with their community (especially children and young people) to understand and map a specific complex problem systems (including factors and relationships that impacts on that complex problem) to then develop community led action that could influence and/or create change within that system. The reason I'm taking this course is to find ways to strengthen the implementation of community led action/initiatives over time and long term.” (366)

## 4 Conclusions

This environmental scan shows that, although training opportunities in IS have increased across North America, they remain largely focused on an academic research audience and on researchers. This may further widen the gap between IS research and practice (17). As a result, IS training programs may not be adequately reaching the wider audience seeking to implement and sustain evidence-based practices across various systems. While numerous programs exist, most are tailored for researchers and graduate students, offering a few spots for non-academic participants. Some programs have fairly strict inclusion criteria, specifically having a “thing” backed by extensive evidence; for many people working to address significant challenges in the system, they are often looking to implementation guidelines, policies, and new practices in topic areas that do not have available evidence-based programs. There is a limited outreach to the sectors and roles where implementation challenges are most significant, such as healthcare delivery, community and social services, and government organizations.

One of the findings that emerged from the environmental scan was the overall reach of current IS training programs. Almost half of the identified programs (47.4%) enroll relatively small cohorts of fewer than 10 participants per cohort. Excluding the two programs that train more than 100 participants annually, the combined maximum estimated enrollment across the remaining 55 programs is approximately 1,420 individuals per year—even when assuming the highest possible enrollment within each cohort's reported range. Considering the number of implementation support practitioners, organizational leaders, intermediaries, and system actors responsible for implementing, spreading, and sustaining evidence-based programs and practices across healthcare, public health, education, social services, and other human service sectors, this level of reach remains limited. The mismatch between the supply of capacity building initiatives and implementation practice needs and demand underscores a critical gap: while training offerings have grown, their overall scale and accessibility remain insufficient to meaningfully support system-wide implementation efforts. Internationally, IS capacity building efforts may also offer useful lessons for expanding training beyond academic audiences. Notably, Viglione and colleagues identified a lack of programs in low- and middle-income countries, underscoring that access gaps are not unique to non-academic audiences in North America but reflect a broader global equity challenge in IS capacity building (3).

Data from The Center for Implementation's courses further reinforces the diversity of professionals across sectors who are interested in building IS capacity. These professionals hold a variety of roles, including organizational leadership, system navigation, clinical service delivery, evaluation, and applied research. These participants described engaging in a wide spectrum of implementation activities and expressed clear motivation to apply IS approaches to ongoing change efforts. These findings highlight that the interest in IS extends beyond academia and that professionals in service delivery, community organizations, and government are seeking out IS training that they can apply to their work and contexts.

Taken together, these findings highlight opportunities to enhance IS capacity building efforts. As the goal of IS is to expand the use of evidence in practice, if we want to scale evidence-based programs, policies, and practices, there is a need for scalable IS training programs to equip a workforce with competencies to implement evidence (26–29). Related to competencies, there is a need to expand on which competencies are covered in IS trainings to have more training programs address competencies related to building implementation infrastructure, navigating system-level complexity, de-implementation, and supporting collaborations across organizations in the implementation system (27). The training programs were only asked about IS topics and were not asked to identify which implementation support competencies were being addressed. More work is needed to understand which implementation support competencies are being addressed (20, 28) and likely to build more training programs that will address those competencies.

There is also a growing interest among non-IS researchers, i.e., implementation support practitioners to learn how to apply IS to their work. These are academics who are not interested in being implementation researchers but applying IS to their work—similar to how implementation support practitioners might apply IS. There is a need to unpack the similarities and differences between this audience and aspiring implementation researchers and implementation support practitioners.

Finally, the field may benefit from greater intentional alignment between academic and applied practice-oriented training. It is not necessarily in the best interest of all participants for everyone to receive the same training, since there are different competencies for implementation research and practice, but there are opportunities for elements of shared curricula, co-designed resources, and practice-based collaboration and mentorship. Another question is whether practice-focused education continues to be delivered through these same institutions; or, to truly meet people's needs alternate training spaces may need to be carved out. For example, alternative programs can focus on competencies for synthesis and translation, support, and implementation delivery functions. Currently, trainings are often too specific or must be broad to bridge gaps, with little consideration of the role people play in implementation.

This study includes several limitations. Survey data were self-reported and may not fully capture the programs or evolution of those programs. We focused exclusively on English training opportunities in North America; therefore, the findings do not reflect what is happening globally and there is the possibility that non-English programs (e.g., French programs in Canada and Spanish programs in Mexico) were excluded; the number of excluded programs was not reported. Programs were categorized by host country; however, this may not reflect the geographic reach of participants, particularly for virtual and hybrid programs. Only currently available publicly posted training programs were surveyed; programs that have been terminated, one-time programs, and employer-specific training programs were excluded. Qualitative data underwent rapid coding and were based on written reflections rather than interviews. Although this study did not directly assess workforce demand or competency needs, the data highlight opportunities for future research to examine how well existing trainings meets the needs of those in implementation practice. Whereas many training programs focus on the needs of the implementation research community, there may be a large audience of people who are less interested in research and more interested in solving public health or health care implementation challenges.

The field has made substantial progress in building IS training programs, yet critical gaps remain. There is a need to broaden access by developing and investing in training models that can prepare a larger and more diverse implementation workforce, expanding the predominant models to reach implementation support practitioners, intermediaries, and applied researchers across sectors. At the same time, training efforts should differentiate and intentionally teach both implementation research competencies and implementation support/practice competencies (for facilitating change in practice), ensuring that learning is relevant and actionable for varied roles. Future research should explore validated approaches to identify implementation support practitioners' status as a functional role rather than a job title, since existing demographic categorizations (e.g., professional sector) may not fully capture individuals performing this work. Strengthening access, aligning training content with role-specific competencies, and scaling delivery models will be essential for supporting the widespread and sustained use of evidence in real-world systems and advancing the core goals of IS.

## Data Availability

The data analyzed in this study is subject to the following licenses/restrictions: Available upon request. Requests to access these datasets should be directed to hbegna@stanford.edu.

## References

[1] Brownson RC Cabassa LJ Drake BF Shelton RC. Closing the gap: advancing implementation science through training and capacity building. Implement Sci. (2024) 19(1):46. 10.1186/s13012-024-01371-x 38961482 PMC11223366

[2] Chambers DA Emmons KM. Navigating the field of implementation science towards maturity: challenges and opportunities. Implement Sci. (2024) 19(1):26. 10.1186/s13012-024-01352-0 38481286 PMC10936041

[3] Viglione C Stadnick NA Birenbaum B Fang O Cakici JA Aarons GA. A systematic review of dissemination and implementation science capacity building programs around the globe. Implement Sci Commun. (2023) 4(1):1. 10.1186/s43058-023-00405-7 36973832 PMC10041476

[4] Baumann AA Adams DR Baldwin L-M Tabak RG Malone S Kepper MM. A mapping review and critique of the literature on translation, dissemination, and implementation capacity building initiatives for different audiences. Implement Sci Commun. (2025) 6(1):34. 10.1186/s43058-025-00717-w 40181484 PMC11970029

[5] Davis R D’Lima D. Building capacity in dissemination and implementation science: a systematic review of the academic literature on teaching and training initiatives. Implement Sci. (2020) 15(1):97. 10.1186/s13012-020-01051-6 33126909 PMC7597006

[6] King O West E Alston L Beks H Callisaya M Huggins CE. Models and approaches for building knowledge translation capacity and capability in health services: a scoping review. Implement Sci. (2024) 19(1):7. 10.1186/s13012-024-01336-0 38287351 PMC10823722

[7] Chambers DA Proctor EK Brownson RC Straus SE. Mapping training needs for dissemination and implementation research: lessons from a synthesis of existing D&I research training programs. Transl Behav Med. (2017) 7(3):593–601. 10.1007/s13142-016-0399-3 27030472 PMC5645270

[8] Brownson RC Jacob RR Carothers BJ Chambers DA Colditz GA Emmons KM. Building the next generation of researchers: mentored training in dissemination and implementation science. Acad Med. (2021) 96(1):86–92. 10.1097/ACM.0000000000003750 32941251 PMC7769184

[9] Padek M Mir N Jacob RR Chambers DA Dobbins M Emmons KM. Training scholars in dissemination and implementation research for cancer prevention and control: a mentored approach. Implement Sci. (2018) 13(1):18. 10.1186/s13012-018-0711-3 29357876 PMC5778694

[10] Proctor EK Chambers DA. Training in dissemination and implementation research: a field-wide perspective. Transl Behav Med. (2017) 7(3):624–35. 10.1007/s13142-016-0406-8 27142266 PMC5645271

[11] Huebschmann AG Johnston S Davis R Kwan BM Geng E Haire-Joshu D. Promoting rigor and sustainment in implementation science capacity building programs: a multi-method study. Implement Res Pract. (2022) 3:26334895221146261. 10.1177/26334895221146261 37091073 PMC9924281

[12] Vinson CA Clyne M Cardoza N Emmons KM. Building capacity: a cross-sectional evaluation of the US training institute for dissemination and implementation research in health. Implement Sci. (2019) 14(1):97. 10.1186/s13012-019-0947-6 31752914 PMC6869199

[13] Albers B Metz A Burke K. Implementation support practitioners—a proposal for consolidating a diverse evidence base. BMC Health Serv Res. (2020) 20(1):368. 10.1186/s12913-020-05145-1 32357877 PMC7193379

[14] Buchanan GJR Filiatreau LM Moore JE. Organizing the dissemination and implementation field: who are we, what are we doing, and how should we do it? Implement Sci Commun. (2024) 5(1):38. 10.1186/s43058-024-00572-1 38605425 PMC11007902

[15] Moore JE Rashid S Park JS Khan S Straus SE. Longitudinal evaluation of a course to build core competencies in implementation practice. Implement Sci. (2018) 13(1):106. 10.1186/s13012-018-0800-3 30081921 PMC6080520

[16] Rapport F Smith J Hutchinson K Clay-Williams R Churruca K Bierbaum M. Too much theory and not enough practice? The challenge of implementation science application in healthcare practice. J Eval Clin Pract. (2022) 28(6):991–1002. 10.1111/jep.13600 34268832

[17] Westerlund A Sundberg L Nilsen P. Implementation of implementation science knowledge: the research-practice gap paradox. Worldviews Evid Based Nurs. (2019) 16(5):332–4. 10.1111/wvn.12403 31603625 PMC6899530

[18] The Center for Implementation. The center for implementation [internet] (2025). The Center for Implementation. Available online at: https://thecenterforimplementation.com (Accessed November 6, 2025).

[19] Padek M Colditz G Dobbins M Koscielniak N Proctor EK Sales AE. Developing educational competencies for dissemination and implementation research training programs: an exploratory analysis using card sorts. Implement Sci. (2015) 10(1):114. 10.1186/s13012-015-0304-3 26264453 PMC4534127

[20] Khan S Manalili K Moore JE. White paper: core competencies and functions of implementation support practitioners [Internet]. The Center for Implementation (2024). Available online at: https://thecenterforimplementation.com/toolbox/white-paper-core-competencies-and-functions-of-implementation-support-practitioners (Accessed December 5, 2025).

[21] Schultes MT Aijaz M Klug J Fixsen DL. Competences for implementation science: what trainees need to learn and where they learn it. Adv Health Sci Educ Theory Pract. (2021) 26(1):19–35. 10.1007/s10459-020-09969-8 32372393 PMC7900055

[22] Straus SE Brouwers M Johnson D Lavis JN Légaré F Majumdar SR. Core competencies in the science and practice of knowledge translation: description of a Canadian strategic training initiative. Implement Sci. (2011) 6:127. 10.1186/1748-5908-6-127 22152223 PMC3292943

[23] Astorino JA Kerch S Pratt-Chapman ML. Building implementation science capacity among practitioners of cancer control: development of a pilot training curriculum. Cancer Causes Control. (2022) 33(9):1181–91. 10.1007/s10552-022-01604-8 35842850 PMC9534372

[24] Taylor B Henshall C Kenyon S Litchfield I Greenfield S. Can rapid approaches to qualitative analysis deliver timely, valid findings to clinical leaders? A mixed methods study comparing rapid and thematic analysis. BMJ Open. (2018) 8(10):e019993. 10.1136/bmjopen-2017-019993 30297341 PMC6194404

[25] Kowalski CP Nevedal AL Finley EP Young JP Lewinski AA Midboe AM. Planning for and assessing rigor in rapid qualitative analysis (PARRQA): a consensus-based framework for designing, conducting, and reporting. Implement Sci. (2024) 19(1):71. 10.1186/s13012-024-01397-1 39394597 PMC11468362

[26] Fleming WO Apostolico AA Mullenix AJ Starr K Margolis L. Putting implementation science into practice: lessons from the creation of the National Maternal and Child Health Workforce Development Center. Matern Child Health J. (2019) 23(6):722–32. 10.1007/s10995-018-02697-x 30684106

[27] Leppin AL Baumann AA Fernandez ME Rudd BN Stevens KR Warner DO. Teaching for implementation: a framework for building implementation research and practice capacity within the translational science workforce. J Clin Transl Sci. (2021) 5(1):e147. 10.1017/cts.2021.809 34527287 PMC8411269

[28] Metz A Jensen T Farley A Boaz A Bartley L Villodas M. Building trusting relationships to support implementation: a proposed theoretical model. Front Health Serv. (2022) 2:894599. 10.3389/frhs.2022.894599 36925800 PMC10012819

[29] Segal L Leach MJ. An evidence-based health workforce model for primary and community care. Implement Sci. (2011) 6(1):93. 10.1186/1748-5908-6-93 21819608 PMC3163196

