# Supplementary material for: Mapping the landscape of implementation science training: gaps and opportunities to strengthen capacity for implementation research and practice
Source: Front Health Serv. 2026 Sep 22;6:1787246. doi: 10.3389/frhs.2026.1787246 (PMC13638569; doi:10.3389/frhs.2026.1787246)
Supplement: Supplementary file 1 [file Table1.docx]

**Supplemental Table 1: Survey responses of 57 identified implementation science training programs in North America**

| **Institution** | **Program Name** | **Website** | **Location** | **Training Level** | **Admission Frequency** | **Cost** | **Cohort Size** | **Duration** | **Audeince(s)** | **Competencies** | **Topic Specificity** |
| --- | --- | --- | --- | --- | --- | --- | --- | --- | --- | --- | --- |
| **Alberta SPOR SUPPORT Unit** | Implementation Science in Patient-Oriented Research Certificate | <https://absporu.ca/certificate-course/implementation-science-in-patient-oriented-research-certificate/> | Canada, Remote (online) | Certificate program, Professional development workshop / training | Annually | $300 CAD (residents)  $1,500 CAD (non-residents) | 11-20 | A few months | Academia (ie. faculty, researchers, students, postdoctoral fellows), Human services professionals, Policy makers, Public health professionals, Research institutes | Foundations of Implementation Science: Defining key terms, distinguishing implementation science from related fields, and explaining its purpose; Frameworks and Theories: Using models, frameworks, and planning tools to guide implementation and sustain change; Assessing Context and Readiness: Identifying what to implement and evaluating setting characteristics, capacity, and barriers; Stakeholder and Community Engagement: Involving partners, communities, and end users through participatory and collaborative approaches; Implementation Strategies: Selecting, tailoring, and applying strategies to improve implementation and scale-up; Measurement and Outcomes: Measuring relevant outcomes and identifying gaps in existing measures | Patient-oriented research |
| **Boston University** | Center for Implementation and Improvement Sciences (CIIS) Fellowship | <https://sites.bu.edu/ciis/> | United States | Fellowship | Annually | Funded | 1-10 | More than one year | Academia (ie. faculty, researchers, students, postdoctoral fellows) | Foundations of Implementation Science: Defining key terms, distinguishing implementation science from related fields, and explaining its purpose; Frameworks and Theories: Using models, frameworks, and planning tools to guide implementation and sustain change; Assessing Context and Readiness: Identifying what to implement and evaluating setting characteristics, capacity, and barriers; Stakeholder and Community Engagement: Involving partners, communities, and end users through participatory and collaborative approaches; Adaptation and Fidelity: Balancing fidelity to interventions while making appropriate adaptations; Implementation Strategies: Selecting, tailoring, and applying strategies to improve implementation and scale-up; Measurement and Outcomes: Measuring relevant outcomes and identifying gaps in existing measures; Study Design and Methods: Designing and analyzing implementation studies using mixed methods and systems thinking; Economic and Policy Considerations: Addressing costs, incentives, and policy influences on implementation; Capacity Building and Team Composition: Building the skills, roles, and infrastructure needed for implementation work; De-implementation: Scaling back or discontinuing ineffective or low-value practices | Individual's research concentration |
| **Cancer Prevention and Control Research Network** | CPCRN Scholars Program Training program | <https://cpcrn.org/scholars> | United States | Graduate level (ie. MPH, MS, MSc, MD, PhD, DrPH, DSW, EdD, PsyD, JD), Post-doctoral, Fellowship, Professional development workshop / training | Annually | Funded | 11-20 | 6 months - 1 year | Academia (ie. faculty, researchers, students, postdoctoral fellows), Community organizations, Healthcare institutions, Healthcare providers, Public health professionals | Foundations of Implementation Science: Defining key terms, distinguishing implementation science from related fields, and explaining its purpose; Frameworks and Theories: Using models, frameworks, and planning tools to guide implementation and sustain change; Assessing Context and Readiness: Identifying what to implement and evaluating setting characteristics, capacity, and barriers; Stakeholder and Community Engagement: Involving partners, communities, and end users through participatory and collaborative approaches; Implementation Strategies: Selecting, tailoring, and applying strategies to improve implementation and scale-up; Measurement and Outcomes: Measuring relevant outcomes and identifying gaps in existing measures; Study Design and Methods: Designing and analyzing implementation studies using mixed methods and systems thinking; Capacity Building and Team Composition: Building the skills, roles, and infrastructure needed for implementation work | Cancer |
| **Center for Dissemination & Implementation at Stanford (C-DIAS)** | C-DIAS Fellowship in Addiction D&I Science | <https://www.c-dias.org/educational-programs/c-dias-fellowship-in-addiction-di/> | United States | Fellowship | Annually | Funded | 1-10 | More than one year | Academia (ie. faculty, researchers, students, postdoctoral fellows), Healthcare providers, Research institutes | Foundations of Implementation Science: Defining key terms, distinguishing implementation science from related fields, and explaining its purpose; Frameworks and Theories: Using models, frameworks, and planning tools to guide implementation and sustain change; Stakeholder and Community Engagement: Involving partners, communities, and end users through participatory and collaborative approaches; Measurement and Outcomes: Measuring relevant outcomes and identifying gaps in existing measures; Study Design and Methods: Designing and analyzing implementation studies using mixed methods and systems thinking | Addiction health services |
| **Collaborative for Implementation Practice (CIPPRO)** | Certificate Program in Implementation Practice | <https://cippro.wpengine.com/certificate/> | Remote (online) | Certificate program | Biannually (twice per year) | $1,800 | 51-100 | 1 - 7 days | Academia (ie. faculty, researchers, students, postdoctoral fellows), Community organizations, Education sector professionals, Funding agencies / Government institutions, Healthcare institutions, Healthcare providers, Human services professionals, Policy makers, Public health professionals, Research institutes | Foundations of Implementation Science: Defining key terms, distinguishing implementation science from related fields, and explaining its purpose; Frameworks and Theories: Using models, frameworks, and planning tools to guide implementation and sustain change; Assessing Context and Readiness: Identifying what to implement and evaluating setting characteristics, capacity, and barriers; Stakeholder and Community Engagement: Involving partners, communities, and end users through participatory and collaborative approaches; Adaptation and Fidelity: Balancing fidelity to interventions while making appropriate adaptations; Implementation Strategies: Selecting, tailoring, and applying strategies to improve implementation and scale-up; Measurement and Outcomes: Measuring relevant outcomes and identifying gaps in existing measures; Economic and Policy Considerations: Addressing costs, incentives, and policy influences on implementation; Capacity Building and Team Composition: Building the skills, roles, and infrastructure needed for implementation work | None |
| **Columbia University Mailman School of Public Health** | Global HIV Implementation Science Fellowship | <https://icap.columbia.edu/student-resources-archive/global-hiv-implementation-science-research-training-fellowship/> | United States | Graduate level (ie. MPH, MS, MSc, MD, PhD, DrPH, DSW, EdD, PsyD, JD) | Annually | Funded | 1-10 | More than one year | Academia (ie. faculty, researchers, students, postdoctoral fellows) | Foundations of Implementation Science: Defining key terms, distinguishing implementation science from related fields, and explaining its purpose; Frameworks and Theories: Using models, frameworks, and planning tools to guide implementation and sustain change; Assessing Context and Readiness: Identifying what to implement and evaluating setting characteristics, capacity, and barriers; Stakeholder and Community Engagement: Involving partners, communities, and end users through participatory and collaborative approaches; Adaptation and Fidelity: Balancing fidelity to interventions while making appropriate adaptations; Implementation Strategies: Selecting, tailoring, and applying strategies to improve implementation and scale-up; Measurement and Outcomes: Measuring relevant outcomes and identifying gaps in existing measures; Study Design and Methods: Designing and analyzing implementation studies using mixed methods and systems thinking; De-implementation: Scaling back or discontinuing ineffective or low-value practices | HIV |
| **Dartmouth College** | Master of Science in Implementation Science | <https://healthsciences.dartmouth.edu/education/degree-programs/master-science-implementation-science/program-overview> | Remote (online) | Graduate level (ie. MPH, MS, MSc, MD, PhD, DrPH, DSW, EdD, PsyD, JD), Post-doctoral, Fellowship, Professional development workshop / training | Annually | $59,190 | 21-50 | 6 months - 1 year | Academia (ie. faculty, researchers, students, postdoctoral fellows), Community organizations, Education sector professionals, Funding agencies / Government institutions, Healthcare institutions, Healthcare providers, Human services professionals, Policy makers, Public health professionals, Research institutes | Foundations of Implementation Science: Defining key terms, distinguishing implementation science from related fields, and explaining its purpose; Frameworks and Theories: Using models, frameworks, and planning tools to guide implementation and sustain change; Assessing Context and Readiness: Identifying what to implement and evaluating setting characteristics, capacity, and barriers; Stakeholder and Community Engagement: Involving partners, communities, and end users through participatory and collaborative approaches; Adaptation and Fidelity: Balancing fidelity to interventions while making appropriate adaptations; Implementation Strategies: Selecting, tailoring, and applying strategies to improve implementation and scale-up; Measurement and Outcomes: Measuring relevant outcomes and identifying gaps in existing measures; Study Design and Methods: Designing and analyzing implementation studies using mixed methods and systems thinking; Economic and Policy Considerations: Addressing costs, incentives, and policy influences on implementation; Capacity Building and Team Composition: Building the skills, roles, and infrastructure needed for implementation work; De-implementation: Scaling back or discontinuing ineffective or low-value practices | None |
| **Emory University** | ACCESS Fellowship in Implementation Science | <https://med.emory.edu/departments/pediatrics/divisions/autism/fellow/implementation-science.html> | United States | Undergraduate level (ie. BA, BS), Fellowship | Annually | Funded | 1-10 | More than one year | Academia (ie. faculty, researchers, students, postdoctoral fellows), Education sector professionals, Healthcare institutions, Healthcare providers, Research institutes | Foundations of Implementation Science: Defining key terms, distinguishing implementation science from related fields, and explaining its purpose; Frameworks and Theories: Using models, frameworks, and planning tools to guide implementation and sustain change; Assessing Context and Readiness: Identifying what to implement and evaluating setting characteristics, capacity, and barriers; Stakeholder and Community Engagement: Involving partners, communities, and end users through participatory and collaborative approaches; Adaptation and Fidelity: Balancing fidelity to interventions while making appropriate adaptations; Implementation Strategies: Selecting, tailoring, and applying strategies to improve implementation and scale-up; Measurement and Outcomes: Measuring relevant outcomes and identifying gaps in existing measures; Study Design and Methods: Designing and analyzing implementation studies using mixed methods and systems thinking; Economic and Policy Considerations: Addressing costs, incentives, and policy influences on implementation; Capacity Building and Team Composition: Building the skills, roles, and infrastructure needed for implementation work | Autism, mental health |
| **Harvard University T.H. Chan School of Public Health** | The HIV Implementation Science (HIS) Training Program | <https://hsph.harvard.edu/fellowship-special-program/the-hiv-implementation-science-his-training-program/> | United States | Graduate level (ie. MPH, MS, MSc, MD, PhD, DrPH, DSW, EdD, PsyD, JD), Post-doctoral | Biannually (twice per year) | Funded | 1-10 | 6 months - 1 year | Academia (ie. faculty, researchers, students, postdoctoral fellows), Community organizations, Public health professionals | Foundations of Implementation Science: Defining key terms, distinguishing implementation science from related fields, and explaining its purpose; Frameworks and Theories: Using models, frameworks, and planning tools to guide implementation and sustain change; Assessing Context and Readiness: Identifying what to implement and evaluating setting characteristics, capacity, and barriers; Stakeholder and Community Engagement: Involving partners, communities, and end users through participatory and collaborative approaches; Adaptation and Fidelity: Balancing fidelity to interventions while making appropriate adaptations; Implementation Strategies: Selecting, tailoring, and applying strategies to improve implementation and scale-up; Measurement and Outcomes: Measuring relevant outcomes and identifying gaps in existing measures; Study Design and Methods: Designing and analyzing implementation studies using mixed methods and systems thinking; Economic and Policy Considerations: Addressing costs, incentives, and policy influences on implementation; Capacity Building and Team Composition: Building the skills, roles, and infrastructure needed for implementation work | HIV |
| **Indiana University** | Certificate in Innovation and Implementation Science | <https://hii.iu.edu/education/graduate-certificate.html> | United States | Graduate level (ie. MPH, MS, MSc, MD, PhD, DrPH, DSW, EdD, PsyD, JD), Post-doctoral, Fellowship, Certificate program | Annually | $19,852.50 | 1-10 | 6 months - 1 year | Academia (ie. faculty, researchers, students, postdoctoral fellows), Community organizations, Education sector professionals, Funding agencies / Government institutions, Healthcare institutions, Healthcare providers, Human services professionals, Policy makers, Public health professionals, Research institutes | Frameworks and Theories: Using models, frameworks, and planning tools to guide implementation and sustain change; Assessing Context and Readiness: Identifying what to implement and evaluating setting characteristics, capacity, and barriers; Stakeholder and Community Engagement: Involving partners, communities, and end users through participatory and collaborative approaches; Capacity Building and Team Composition: Building the skills, roles, and infrastructure needed for implementation work | None |
| **Johns Hopkins University** | DrPH concentration, Implementation Science | <https://publichealth.jhu.edu/academics/drph/curriculum/concentration-in-implementation-science> | United States | Graduate level (ie. MPH, MS, MSc, MD, PhD, DrPH, DSW, EdD, PsyD, JD) | Annually | $91,456 | 1-10 | More than one year | Academia (ie. faculty, researchers, students, postdoctoral fellows), Community organizations, Education sector professionals, Funding agencies / Government institutions, Healthcare institutions, Healthcare providers, Human services professionals, Policy makers, Public health professionals | Foundations of Implementation Science: Defining key terms, distinguishing implementation science from related fields, and explaining its purpose; Frameworks and Theories: Using models, frameworks, and planning tools to guide implementation and sustain change; Assessing Context and Readiness: Identifying what to implement and evaluating setting characteristics, capacity, and barriers; Stakeholder and Community Engagement: Involving partners, communities, and end users through participatory and collaborative approaches; Adaptation and Fidelity: Balancing fidelity to interventions while making appropriate adaptations; Implementation Strategies: Selecting, tailoring, and applying strategies to improve implementation and scale-up; Measurement and Outcomes: Measuring relevant outcomes and identifying gaps in existing measures; Study Design and Methods: Designing and analyzing implementation studies using mixed methods and systems thinking; Economic and Policy Considerations: Addressing costs, incentives, and policy influences on implementation; Capacity Building and Team Composition: Building the skills, roles, and infrastructure needed for implementation work | Individual's research concentration |
| **Johns Hopkins University** | Certificate in Implementation Science and Research Practice | <https://e-catalogue.jhu.edu/public-health/certificates/implementation-science-research-practice/> | United States | Certificate program | Rolling enrollment | $25,722 | 1-10 | More than one year | Academia (ie. faculty, researchers, students, postdoctoral fellows), Community organizations, Education sector professionals, Funding agencies / Government institutions, Healthcare institutions, Healthcare providers, Human services professionals, Policy makers, Public health professionals, Research institutes | Foundations of Implementation Science: Defining key terms, distinguishing implementation science from related fields, and explaining its purpose; Frameworks and Theories: Using models, frameworks, and planning tools to guide implementation and sustain change; Assessing Context and Readiness: Identifying what to implement and evaluating setting characteristics, capacity, and barriers; Stakeholder and Community Engagement: Involving partners, communities, and end users through participatory and collaborative approaches; Adaptation and Fidelity: Balancing fidelity to interventions while making appropriate adaptations; Implementation Strategies: Selecting, tailoring, and applying strategies to improve implementation and scale-up; Measurement and Outcomes: Measuring relevant outcomes and identifying gaps in existing measures; Study Design and Methods: Designing and analyzing implementation studies using mixed methods and systems thinking; Economic and Policy Considerations: Addressing costs, incentives, and policy influences on implementation; Capacity Building and Team Composition: Building the skills, roles, and infrastructure needed for implementation work | None |
| **Knowledge Translation (KT) Canada** | Summer Institute Workshop | <https://ktcanada.org/event/kt-canada-summer-institute-2025/> | Canada | Certificate program, Professional development workshop / training | Annually | $1,075 CAD | 21-50 | 1 - 7 days | Academia (ie. faculty, researchers, students, postdoctoral fellows) | Foundations of Implementation Science: Defining key terms, distinguishing implementation science from related fields, and explaining its purpose; Frameworks and Theories: Using models, frameworks, and planning tools to guide implementation and sustain change; Assessing Context and Readiness: Identifying what to implement and evaluating setting characteristics, capacity, and barriers; Stakeholder and Community Engagement: Involving partners, communities, and end users through participatory and collaborative approaches; Adaptation and Fidelity: Balancing fidelity to interventions while making appropriate adaptations; Implementation Strategies: Selecting, tailoring, and applying strategies to improve implementation and scale-up; Measurement and Outcomes: Measuring relevant outcomes and identifying gaps in existing measures; Study Design and Methods: Designing and analyzing implementation studies using mixed methods and systems thinking; Capacity Building and Team Composition: Building the skills, roles, and infrastructure needed for implementation work | None |
| **Knowledge Translation Program** | Practicing Knowledge Translation | <https://knowledgetranslation.net/event/practicing-knowledge-translation/> | Canada, Remote (online) | Certificate program, Professional development workshop / training | Annually | $2,150 CAD | 11-20 | 1 - 7 days | Academia (ie. faculty, researchers, students, postdoctoral fellows), Community organizations, Healthcare institutions, Healthcare providers, Human services professionals, Policy makers, Public health professionals, Research institutes | Foundations of Implementation Science: Defining key terms, distinguishing implementation science from related fields, and explaining its purpose; Frameworks and Theories: Using models, frameworks, and planning tools to guide implementation and sustain change; Assessing Context and Readiness: Identifying what to implement and evaluating setting characteristics, capacity, and barriers; Stakeholder and Community Engagement: Involving partners, communities, and end users through participatory and collaborative approaches; Adaptation and Fidelity: Balancing fidelity to interventions while making appropriate adaptations; Implementation Strategies: Selecting, tailoring, and applying strategies to improve implementation and scale-up; Measurement and Outcomes: Measuring relevant outcomes and identifying gaps in existing measures; Study Design and Methods: Designing and analyzing implementation studies using mixed methods and systems thinking; Capacity Building and Team Composition: Building the skills, roles, and infrastructure needed for implementation work | None |
| **Northwestern University** | Practitioner-Oriented Implementation Science Education & Training (POISE) | <https://hivimpsci.northwestern.edu/project/practitioner-oriented-implementation-science-education-training/> | United States, Remote (online) | Professional development workshop / training | Annually | Funded | 1-10 | A few months | Healthcare providers, Human services professionals, Public health professionals | Foundations of Implementation Science: Defining key terms, distinguishing implementation science from related fields, and explaining its purpose; Frameworks and Theories: Using models, frameworks, and planning tools to guide implementation and sustain change; Assessing Context and Readiness: Identifying what to implement and evaluating setting characteristics, capacity, and barriers; Implementation Strategies: Selecting, tailoring, and applying strategies to improve implementation and scale-up; Measurement and Outcomes: Measuring relevant outcomes and identifying gaps in existing measures | HIV |
| **Northwestern University** | Implementation Science Academy | <https://www.mss.northwestern.edu/education/methodological-training-institutes.html> | United States | Professional development workshop / training | Annually | $3,000 | 11-20 | 1 - 7 days | Academia (ie. faculty, researchers, students, postdoctoral fellows), Education sector professionals, Healthcare providers, Public health professionals | Foundations of Implementation Science: Defining key terms, distinguishing implementation science from related fields, and explaining its purpose; Frameworks and Theories: Using models, frameworks, and planning tools to guide implementation and sustain change; Assessing Context and Readiness: Identifying what to implement and evaluating setting characteristics, capacity, and barriers; Stakeholder and Community Engagement: Involving partners, communities, and end users through participatory and collaborative approaches; Adaptation and Fidelity: Balancing fidelity to interventions while making appropriate adaptations; Implementation Strategies: Selecting, tailoring, and applying strategies to improve implementation and scale-up; Measurement and Outcomes: Measuring relevant outcomes and identifying gaps in existing measures; Study Design and Methods: Designing and analyzing implementation studies using mixed methods and systems thinking; Economic and Policy Considerations: Addressing costs, incentives, and policy influences on implementation; De-implementation: Scaling back or discontinuing ineffective or low-value practices | None |
| **Northwestern University** | Center for Dissemination & Implementation Science (CDIS) Training Institute | <https://www.feinberg.northwestern.edu/sites/cdis/training-support/index.html> | United States | Professional development workshop / training | Annually | Funded | 11-20 | 1 - 7 days | Academia (ie. faculty, researchers, students, postdoctoral fellows), Education sector professionals, Healthcare providers, Public health professionals | Foundations of Implementation Science: Defining key terms, distinguishing implementation science from related fields, and explaining its purpose; Frameworks and Theories: Using models, frameworks, and planning tools to guide implementation and sustain change; Assessing Context and Readiness: Identifying what to implement and evaluating setting characteristics, capacity, and barriers; Stakeholder and Community Engagement: Involving partners, communities, and end users through participatory and collaborative approaches; Adaptation and Fidelity: Balancing fidelity to interventions while making appropriate adaptations; Implementation Strategies: Selecting, tailoring, and applying strategies to improve implementation and scale-up; Measurement and Outcomes: Measuring relevant outcomes and identifying gaps in existing measures; Study Design and Methods: Designing and analyzing implementation studies using mixed methods and systems thinking; Economic and Policy Considerations: Addressing costs, incentives, and policy influences on implementation; De-implementation: Scaling back or discontinuing ineffective or low-value practices | None |
| **Northwestern University** | CDIS Reading Course | <https://www.feinberg.northwestern.edu/sites/cdis/training-support/implementation-science-reading-course.html> | United States | Post-doctoral, Fellowship, Professional development workshop / training | Annually | Funded | 51-100 | A few months | Academia (ie. faculty, researchers, students, postdoctoral fellows) | Foundations of Implementation Science: Defining key terms, distinguishing implementation science from related fields, and explaining its purpose; Frameworks and Theories: Using models, frameworks, and planning tools to guide implementation and sustain change; Assessing Context and Readiness: Identifying what to implement and evaluating setting characteristics, capacity, and barriers; Stakeholder and Community Engagement: Involving partners, communities, and end users through participatory and collaborative approaches; Adaptation and Fidelity: Balancing fidelity to interventions while making appropriate adaptations; Implementation Strategies: Selecting, tailoring, and applying strategies to improve implementation and scale-up; Measurement and Outcomes: Measuring relevant outcomes and identifying gaps in existing measures; Study Design and Methods: Designing and analyzing implementation studies using mixed methods and systems thinking | None |
| **Northwestern University** | MPH concentration, Implementation Science in Public Health and Medicine | <https://www.feinberg.northwestern.edu/sites/mph/degree-options/mph/curriculum.html> | Remote (online) | Graduate level (ie. MPH, MS, MSc, MD, PhD, DrPH, DSW, EdD, PsyD, JD) | Annually | $54,720 | 21-50 | 6 months - 1 year | Academia (ie. faculty, researchers, students, postdoctoral fellows) | Foundations of Implementation Science: Defining key terms, distinguishing implementation science from related fields, and explaining its purpose; Frameworks and Theories: Using models, frameworks, and planning tools to guide implementation and sustain change; Assessing Context and Readiness: Identifying what to implement and evaluating setting characteristics, capacity, and barriers; Stakeholder and Community Engagement: Involving partners, communities, and end users through participatory and collaborative approaches; Adaptation and Fidelity: Balancing fidelity to interventions while making appropriate adaptations; Implementation Strategies: Selecting, tailoring, and applying strategies to improve implementation and scale-up; Study Design and Methods: Designing and analyzing implementation studies using mixed methods and systems thinking | None |
| **Northwestern University** | Certificate in Implementation Science in Public Health and Medicine | <https://www.feinberg.northwestern.edu/sites/pph/index.html> | Remote (online) | Certificate program | Annually | $22,000 | 1-10 | 6 months - 1 year | Academia (ie. faculty, researchers, students, postdoctoral fellows), Education sector professionals, Healthcare providers, Public health professionals | Foundations of Implementation Science: Defining key terms, distinguishing implementation science from related fields, and explaining its purpose; Frameworks and Theories: Using models, frameworks, and planning tools to guide implementation and sustain change; Assessing Context and Readiness: Identifying what to implement and evaluating setting characteristics, capacity, and barriers; Stakeholder and Community Engagement: Involving partners, communities, and end users through participatory and collaborative approaches; Adaptation and Fidelity: Balancing fidelity to interventions while making appropriate adaptations; Implementation Strategies: Selecting, tailoring, and applying strategies to improve implementation and scale-up; Measurement and Outcomes: Measuring relevant outcomes and identifying gaps in existing measures; Study Design and Methods: Designing and analyzing implementation studies using mixed methods and systems thinking | None |
| **Penn State Clinical and Translational Science Institute** | Implementation Science Fellowship | <https://ctsi.psu.edu/isc/fellowship/> | United States | Fellowship, Professional development workshop / training | Annually | Funded | 1-10 | 6 months - 1 year | Academia (ie. faculty, researchers, students, postdoctoral fellows), Healthcare providers | Foundations of Implementation Science: Defining key terms, distinguishing implementation science from related fields, and explaining its purpose; Frameworks and Theories: Using models, frameworks, and planning tools to guide implementation and sustain change; Adaptation and Fidelity: Balancing fidelity to interventions while making appropriate adaptations; Implementation Strategies: Selecting, tailoring, and applying strategies to improve implementation and scale-up; Measurement and Outcomes: Measuring relevant outcomes and identifying gaps in existing measures; Study Design and Methods: Designing and analyzing implementation studies using mixed methods and systems thinking | None |
| **The Baker Center for Children and Families** | Postdoctoral Fellowship in Applied Implementation Science | <https://www.bakercenter.org/postdoc-fellowship> | United States | Post-doctoral | Annually | Funded | 1-10 | 6 months - 1 year | Academia (ie. faculty, researchers, students, postdoctoral fellows) | Foundations of Implementation Science: Defining key terms, distinguishing implementation science from related fields, and explaining its purpose; Frameworks and Theories: Using models, frameworks, and planning tools to guide implementation and sustain change; Assessing Context and Readiness: Identifying what to implement and evaluating setting characteristics, capacity, and barriers; Stakeholder and Community Engagement: Involving partners, communities, and end users through participatory and collaborative approaches; Adaptation and Fidelity: Balancing fidelity to interventions while making appropriate adaptations; Implementation Strategies: Selecting, tailoring, and applying strategies to improve implementation and scale-up; Measurement and Outcomes: Measuring relevant outcomes and identifying gaps in existing measures; Economic and Policy Considerations: Addressing costs, incentives, and policy influences on implementation; Capacity Building and Team Composition: Building the skills, roles, and infrastructure needed for implementation work | Mental health |
| **The Center for Implementation** | Implementation Support Specialist Certificate Program | <https://thecenterforimplementation.com/> | Remote (online) | Certificate program, Professional development workshop / training | Rolling enrollment | $1,800 | 100+ | 6 months - 1 year | Academia (ie. faculty, researchers, students, postdoctoral fellows), Community organizations, Education sector professionals, Funding agencies / Government institutions, Healthcare institutions, Healthcare providers, Human services professionals, Public health professionals, Research institutes | Foundations of Implementation Science: Defining key terms, distinguishing implementation science from related fields, and explaining its purpose; Frameworks and Theories: Using models, frameworks, and planning tools to guide implementation and sustain change; Assessing Context and Readiness: Identifying what to implement and evaluating setting characteristics, capacity, and barriers; Stakeholder and Community Engagement: Involving partners, communities, and end users through participatory and collaborative approaches; Adaptation and Fidelity: Balancing fidelity to interventions while making appropriate adaptations; Implementation Strategies: Selecting, tailoring, and applying strategies to improve implementation and scale-up; Measurement and Outcomes: Measuring relevant outcomes and identifying gaps in existing measures; Capacity Building and Team Composition: Building the skills, roles, and infrastructure needed for implementation work | None |
| **The Hospital for Sick Children’s (SickKids)** | Planning for Implementation Practice (PIP™) Workshop | <https://www.sickkids.ca/en/learning/our-programs/knowledge-translation-program/planning-for-implmentation-practice-pip/> | Canada, Remote (online) | Undergraduate level (ie. BA, BS), Graduate level (ie. MPH, MS, MSc, MD, PhD, DrPH, DSW, EdD, PsyD, JD), Post-doctoral, Fellowship, Professional development workshop / training | Biannually (twice per year) | $700 CAD | 21-50 | 1 - 7 days | Academia (ie. faculty, researchers, students, postdoctoral fellows), Community organizations, Human services professionals, Public health professionals, Research institutes | Frameworks and Theories: Using models, frameworks, and planning tools to guide implementation and sustain change; Assessing Context and Readiness: Identifying what to implement and evaluating setting characteristics, capacity, and barriers; Implementation Strategies: Selecting, tailoring, and applying strategies to improve implementation and scale-up; Measurement and Outcomes: Measuring relevant outcomes and identifying gaps in existing measures | None |
| **The Hospital for Sick Children’s (SickKids)** | Knowledge Translation Professional Certificate (KTPC) | <https://www.sickkids.ca/en/learning/continuing-professional-development/knowledge-translation-training/#ktpc> | Canada, Remote (online) | Undergraduate level (ie. BA, BS), Graduate level (ie. MPH, MS, MSc, MD, PhD, DrPH, DSW, EdD, PsyD, JD), Post-doctoral, Fellowship, Certificate program, Professional development workshop / training | Multiple admissions per year | $2,800 CAD | 21-50 | 1 - 7 days | Academia (ie. faculty, researchers, students, postdoctoral fellows), Community organizations, Funding agencies / Government institutions, Healthcare institutions, Human services professionals, Public health professionals, Research institutes | Foundations of Implementation Science: Defining key terms, distinguishing implementation science from related fields, and explaining its purpose; Implementation Strategies: Selecting, tailoring, and applying strategies to improve implementation and scale-up; Measurement and Outcomes: Measuring relevant outcomes and identifying gaps in existing measures | None |
| **University of Arkansas for Medical Sciences** | Graduate Certificate in Implementation Science | <https://gradschool.uams.edu/prospective-students/programs/graduate-certificate-in-implementation-science> | United States, Remote (online) | Certificate program | Rolling enrollment | $5,712 (resident)  $11,424 (non-resident) | 1-10 | More than one year | Academia (ie. faculty, researchers, students, postdoctoral fellows) | Foundations of Implementation Science: Defining key terms, distinguishing implementation science from related fields, and explaining its purpose; Frameworks and Theories: Using models, frameworks, and planning tools to guide implementation and sustain change; Assessing Context and Readiness: Identifying what to implement and evaluating setting characteristics, capacity, and barriers; Stakeholder and Community Engagement: Involving partners, communities, and end users through participatory and collaborative approaches; Adaptation and Fidelity: Balancing fidelity to interventions while making appropriate adaptations; Implementation Strategies: Selecting, tailoring, and applying strategies to improve implementation and scale-up; Measurement and Outcomes: Measuring relevant outcomes and identifying gaps in existing measures; Study Design and Methods: Designing and analyzing implementation studies using mixed methods and systems thinking; Economic and Policy Considerations: Addressing costs, incentives, and policy influences on implementation; Capacity Building and Team Composition: Building the skills, roles, and infrastructure needed for implementation work; De-implementation: Scaling back or discontinuing ineffective or low-value practices | None |
| **University of Arkansas for Medical Sciences** | Implementation Science Scholar Program / Fellowship | <https://tri.uams.edu/resources-and-services/tri-services/the-center-for-implementation-research/implementation-science-scholar-program/> | United States | Fellowship, Professional development workshop / training | Annually | Funded | 1-10 | More than one year | Healthcare providers | Foundations of Implementation Science: Defining key terms, distinguishing implementation science from related fields, and explaining its purpose; Frameworks and Theories: Using models, frameworks, and planning tools to guide implementation and sustain change; Assessing Context and Readiness: Identifying what to implement and evaluating setting characteristics, capacity, and barriers; Stakeholder and Community Engagement: Involving partners, communities, and end users through participatory and collaborative approaches; Implementation Strategies: Selecting, tailoring, and applying strategies to improve implementation and scale-up; Measurement and Outcomes: Measuring relevant outcomes and identifying gaps in existing measures; De-implementation: Scaling back or discontinuing ineffective or low-value practices | None |
| **University of British Columbia** | SFU-UBC Implementation Science Training Initiative | <https://kx.ubc.ca/programs/isti> | Canada | Fellowship, Professional development workshop / training, Webinar series | Rolling enrollment | Funded | 11-20 | More than one year | Academia (ie. faculty, researchers, students, postdoctoral fellows) | Foundations of Implementation Science: Defining key terms, distinguishing implementation science from related fields, and explaining its purpose; Frameworks and Theories: Using models, frameworks, and planning tools to guide implementation and sustain change; Assessing Context and Readiness: Identifying what to implement and evaluating setting characteristics, capacity, and barriers; Stakeholder and Community Engagement: Involving partners, communities, and end users through participatory and collaborative approaches; Adaptation and Fidelity: Balancing fidelity to interventions while making appropriate adaptations; Implementation Strategies: Selecting, tailoring, and applying strategies to improve implementation and scale-up; Study Design and Methods: Designing and analyzing implementation studies using mixed methods and systems thinking; De-implementation: Scaling back or discontinuing ineffective or low-value practices | None |
| **University of California, San Diego** | DrPH concentration, Health Services Research and Implementation Science | <https://ph.ucsd.edu/phd/public-health/index.html> | United States | Graduate level (ie. MPH, MS, MSc, MD, PhD, DrPH, DSW, EdD, PsyD, JD) | Annually | Funded | 1-10 | More than one year | Academia (ie. faculty, researchers, students, postdoctoral fellows), Education sector professionals, Healthcare providers, Human services professionals, Policy makers, Public health professionals | Foundations of Implementation Science: Defining key terms, distinguishing implementation science from related fields, and explaining its purpose; Frameworks and Theories: Using models, frameworks, and planning tools to guide implementation and sustain change; Assessing Context and Readiness: Identifying what to implement and evaluating setting characteristics, capacity, and barriers; Stakeholder and Community Engagement: Involving partners, communities, and end users through participatory and collaborative approaches; Adaptation and Fidelity: Balancing fidelity to interventions while making appropriate adaptations; Implementation Strategies: Selecting, tailoring, and applying strategies to improve implementation and scale-up; Measurement and Outcomes: Measuring relevant outcomes and identifying gaps in existing measures; Study Design and Methods: Designing and analyzing implementation studies using mixed methods and systems thinking; Economic and Policy Considerations: Addressing costs, incentives, and policy influences on implementation; Capacity Building and Team Composition: Building the skills, roles, and infrastructure needed for implementation work | Health services research |
| **University of California, San Francisco** | Online Certificate in Implementation Science | <https://epibiostat.ucsf.edu/online-certificate-implementation-science> | Remote (online) | Graduate level (ie. MPH, MS, MSc, MD, PhD, DrPH, DSW, EdD, PsyD, JD), Post-doctoral, Fellowship, Certificate program, Professional development workshop / training | Rolling enrollment | $17,400 | 21-50 | More than one year | Academia (ie. faculty, researchers, students, postdoctoral fellows), Community organizations, Funding agencies / Government institutions, Healthcare institutions, Healthcare providers, Human services professionals, Policy makers, Public health professionals, Research institutes | Foundations of Implementation Science: Defining key terms, distinguishing implementation science from related fields, and explaining its purpose; Frameworks and Theories: Using models, frameworks, and planning tools to guide implementation and sustain change; Assessing Context and Readiness: Identifying what to implement and evaluating setting characteristics, capacity, and barriers; Stakeholder and Community Engagement: Involving partners, communities, and end users through participatory and collaborative approaches; Adaptation and Fidelity: Balancing fidelity to interventions while making appropriate adaptations; Implementation Strategies: Selecting, tailoring, and applying strategies to improve implementation and scale-up; Measurement and Outcomes: Measuring relevant outcomes and identifying gaps in existing measures; Study Design and Methods: Designing and analyzing implementation studies using mixed methods and systems thinking; De-implementation: Scaling back or discontinuing ineffective or low-value practices | None |
| **University of California, San Francisco** | MS concentration, Implementation Science Track | <https://epibiostat.ucsf.edu/implementation-science-track> | United States | Graduate level (ie. MPH, MS, MSc, MD, PhD, DrPH, DSW, EdD, PsyD, JD) | Annually | $68,100 | 21-50 | More than one year | Academia (ie. faculty, researchers, students, postdoctoral fellows), Community organizations, Education sector professionals, Funding agencies / Government institutions, Healthcare institutions, Healthcare providers, Human services professionals, Policy makers, Public health professionals, Research institutes | Foundations of Implementation Science: Defining key terms, distinguishing implementation science from related fields, and explaining its purpose; Frameworks and Theories: Using models, frameworks, and planning tools to guide implementation and sustain change; Stakeholder and Community Engagement: Involving partners, communities, and end users through participatory and collaborative approaches; Adaptation and Fidelity: Balancing fidelity to interventions while making appropriate adaptations; Implementation Strategies: Selecting, tailoring, and applying strategies to improve implementation and scale-up; Measurement and Outcomes: Measuring relevant outcomes and identifying gaps in existing measures; Study Design and Methods: Designing and analyzing implementation studies using mixed methods and systems thinking | None |
| **University of California, San Francisco** | (PRISE) Partnerships for Research in Implementation Science for Equity T32 Training Program | <https://prise.ucsf.edu/training/prise-t32/> | United States, Remote (online) | Graduate level (ie. MPH, MS, MSc, MD, PhD, DrPH, DSW, EdD, PsyD, JD), Post-doctoral, Fellowship, Professional development workshop / training | Multiple admissions per year | Funded | 21-50 | 1 - 7 days | Academia (ie. faculty, researchers, students, postdoctoral fellows), Community organizations, Funding agencies / Government institutions, Policy makers, Public health professionals, Research institutes, researchers, clinicians, and public health practitioners | Foundations of Implementation Science: Defining key terms, distinguishing implementation science from related fields, and explaining its purpose; Frameworks and Theories: Using models, frameworks, and planning tools to guide implementation and sustain change; Assessing Context and Readiness: Identifying what to implement and evaluating setting characteristics, capacity, and barriers; Stakeholder and Community Engagement: Involving partners, communities, and end users through participatory and collaborative approaches; Adaptation and Fidelity: Balancing fidelity to interventions while making appropriate adaptations; Implementation Strategies: Selecting, tailoring, and applying strategies to improve implementation and scale-up; Measurement and Outcomes: Measuring relevant outcomes and identifying gaps in existing measures; Study Design and Methods: Designing and analyzing implementation studies using mixed methods and systems thinking | None |
| **University of Colorado Anschutz Medical Campus** | D&I Science Graduate Certificate | <https://medschool.cuanschutz.edu/accords/cores-and-programs/dissemination-implementation-core/d-i-certificate-program> | Remote (online) | Graduate level (ie. MPH, MS, MSc, MD, PhD, DrPH, DSW, EdD, PsyD, JD), Certificate program | Annually | $,7164 (resident)  $17,412 (non-resident) | 11-20 | More than one year | Academia (ie. faculty, researchers, students, postdoctoral fellows), Community organizations, Funding agencies / Government institutions, Healthcare institutions, Healthcare providers, Human services professionals, Public health professionals, Research institutes | Foundations of Implementation Science: Defining key terms, distinguishing implementation science from related fields, and explaining its purpose; Frameworks and Theories: Using models, frameworks, and planning tools to guide implementation and sustain change; Assessing Context and Readiness: Identifying what to implement and evaluating setting characteristics, capacity, and barriers; Stakeholder and Community Engagement: Involving partners, communities, and end users through participatory and collaborative approaches; Adaptation and Fidelity: Balancing fidelity to interventions while making appropriate adaptations; Implementation Strategies: Selecting, tailoring, and applying strategies to improve implementation and scale-up; Measurement and Outcomes: Measuring relevant outcomes and identifying gaps in existing measures; Study Design and Methods: Designing and analyzing implementation studies using mixed methods and systems thinking; Economic and Policy Considerations: Addressing costs, incentives, and policy influences on implementation; Capacity Building and Team Composition: Building the skills, roles, and infrastructure needed for implementation work; De-implementation: Scaling back or discontinuing ineffective or low-value practices | None |
| **University of Colorado Anschutz Medical Campus** | D&I Science for Researchers Workshop | <https://medschool.cuanschutz.edu/accords/cores-and-programs/dissemination-implementation-core/d-i-science-workshops/d-i-science-for-researchers-workshop-2025> | Remote (online) | Undergraduate level (ie. BA, BS), Graduate level (ie. MPH, MS, MSc, MD, PhD, DrPH, DSW, EdD, PsyD, JD), Post-doctoral, Fellowship, Professional development workshop / training | Annually | $500 | 51-100 | 1 - 7 days | Academia (ie. faculty, researchers, students, postdoctoral fellows), Community organizations, Funding agencies / Government institutions, Healthcare institutions, Healthcare providers, Human services professionals, Policy makers, Public health professionals, Research institutes | Foundations of Implementation Science: Defining key terms, distinguishing implementation science from related fields, and explaining its purpose; Frameworks and Theories: Using models, frameworks, and planning tools to guide implementation and sustain change; Assessing Context and Readiness: Identifying what to implement and evaluating setting characteristics, capacity, and barriers; Stakeholder and Community Engagement: Involving partners, communities, and end users through participatory and collaborative approaches; Adaptation and Fidelity: Balancing fidelity to interventions while making appropriate adaptations; Implementation Strategies: Selecting, tailoring, and applying strategies to improve implementation and scale-up; Measurement and Outcomes: Measuring relevant outcomes and identifying gaps in existing measures; Study Design and Methods: Designing and analyzing implementation studies using mixed methods and systems thinking; Economic and Policy Considerations: Addressing costs, incentives, and policy influences on implementation; Capacity Building and Team Composition: Building the skills, roles, and infrastructure needed for implementation work; De-implementation: Scaling back or discontinuing ineffective or low-value practices | None |
| **University of Florida** | Graduate Certificate in Implementation Science | <https://hobi.med.ufl.edu/education/graduate-certificate-in-implementation-science/> | United States | Certificate program | Biannually (twice per year) | $7,150 | 1-10 | More than one year | Academia (ie. faculty, researchers, students, postdoctoral fellows), Healthcare providers, Public health professionals | Foundations of Implementation Science: Defining key terms, distinguishing implementation science from related fields, and explaining its purpose; Frameworks and Theories: Using models, frameworks, and planning tools to guide implementation and sustain change; Stakeholder and Community Engagement: Involving partners, communities, and end users through participatory and collaborative approaches; Implementation Strategies: Selecting, tailoring, and applying strategies to improve implementation and scale-up; Study Design and Methods: Designing and analyzing implementation studies using mixed methods and systems thinking | None |
| **University of Florida** | MS concentration, Health Outcomes and Implementation Science | <https://hobi.med.ufl.edu/education/master-of-science-in-health-outcomes-and-implementation-science/> | United States | Graduate level (ie. MPH, MS, MSc, MD, PhD, DrPH, DSW, EdD, PsyD, JD) | Annually | $25,840 | 1-10 | More than one year | Academia (ie. faculty, researchers, students, postdoctoral fellows) | Foundations of Implementation Science: Defining key terms, distinguishing implementation science from related fields, and explaining its purpose; Frameworks and Theories: Using models, frameworks, and planning tools to guide implementation and sustain change; Assessing Context and Readiness: Identifying what to implement and evaluating setting characteristics, capacity, and barriers; Stakeholder and Community Engagement: Involving partners, communities, and end users through participatory and collaborative approaches; Adaptation and Fidelity: Balancing fidelity to interventions while making appropriate adaptations; Implementation Strategies: Selecting, tailoring, and applying strategies to improve implementation and scale-up; Measurement and Outcomes: Measuring relevant outcomes and identifying gaps in existing measures; Study Design and Methods: Designing and analyzing implementation studies using mixed methods and systems thinking | None |
| **University of Florida** | PhD concentration, Health Outcomes and Implementation Science | <https://hobi.med.ufl.edu/education/phd-in-health-outcomes-and-implementation-science/> | United States | Graduate level (ie. MPH, MS, MSc, MD, PhD, DrPH, DSW, EdD, PsyD, JD) | Annually | Funded | 1-10 | More than one year | Academia (ie. faculty, researchers, students, postdoctoral fellows) | Foundations of Implementation Science: Defining key terms, distinguishing implementation science from related fields, and explaining its purpose; Frameworks and Theories: Using models, frameworks, and planning tools to guide implementation and sustain change; Assessing Context and Readiness: Identifying what to implement and evaluating setting characteristics, capacity, and barriers; Stakeholder and Community Engagement: Involving partners, communities, and end users through participatory and collaborative approaches; Adaptation and Fidelity: Balancing fidelity to interventions while making appropriate adaptations; Implementation Strategies: Selecting, tailoring, and applying strategies to improve implementation and scale-up; Measurement and Outcomes: Measuring relevant outcomes and identifying gaps in existing measures; Study Design and Methods: Designing and analyzing implementation studies using mixed methods and systems thinking | None |
| **University of Maryland, Baltimore** | Implementation Science Certificate Program | <https://graduate.umaryland.edu/research/> | Remote (online) | Graduate level (ie. MPH, MS, MSc, MD, PhD, DrPH, DSW, EdD, PsyD, JD), Certificate program | Rolling enrollment | $9,228 (resident)  $11,928 (non-resident) | 11-20 | More than one year | Academia (ie. faculty, researchers, students, postdoctoral fellows), Community organizations, Education sector professionals, Funding agencies / Government institutions, Healthcare institutions, Healthcare providers, Human services professionals, Policy makers, Public health professionals, Research institutes | Foundations of Implementation Science: Defining key terms, distinguishing implementation science from related fields, and explaining its purpose; Frameworks and Theories: Using models, frameworks, and planning tools to guide implementation and sustain change; Assessing Context and Readiness: Identifying what to implement and evaluating setting characteristics, capacity, and barriers; Stakeholder and Community Engagement: Involving partners, communities, and end users through participatory and collaborative approaches; Adaptation and Fidelity: Balancing fidelity to interventions while making appropriate adaptations; Implementation Strategies: Selecting, tailoring, and applying strategies to improve implementation and scale-up; Measurement and Outcomes: Measuring relevant outcomes and identifying gaps in existing measures; Study Design and Methods: Designing and analyzing implementation studies using mixed methods and systems thinking; Economic and Policy Considerations: Addressing costs, incentives, and policy influences on implementation; Capacity Building and Team Composition: Building the skills, roles, and infrastructure needed for implementation work; De-implementation: Scaling back or discontinuing ineffective or low-value practices | None |
| **University of North Carolina at Chapel Hill** | Interprofessional Graduate Certificate in Improvement Science and Implementation | <https://ed.unc.edu/academics/programs/interprofessional-graduate-certificate-in-improvement-science-and-implementation/> | United States, Remote (online) | Certificate program | Annually | $5,382 (resident)  $14,710 (non-resident) | 11-20 | 6 months - 1 year | Academia (ie. faculty, researchers, students, postdoctoral fellows), Community organizations, Education sector professionals, Healthcare institutions, Healthcare providers, Human services professionals, Public health professionals | Foundations of Implementation Science: Defining key terms, distinguishing implementation science from related fields, and explaining its purpose; Frameworks and Theories: Using models, frameworks, and planning tools to guide implementation and sustain change; Assessing Context and Readiness: Identifying what to implement and evaluating setting characteristics, capacity, and barriers; Stakeholder and Community Engagement: Involving partners, communities, and end users through participatory and collaborative approaches; Adaptation and Fidelity: Balancing fidelity to interventions while making appropriate adaptations; Implementation Strategies: Selecting, tailoring, and applying strategies to improve implementation and scale-up; Measurement and Outcomes: Measuring relevant outcomes and identifying gaps in existing measures; Study Design and Methods: Designing and analyzing implementation studies using mixed methods and systems thinking; Economic and Policy Considerations: Addressing costs, incentives, and policy influences on implementation; Capacity Building and Team Composition: Building the skills, roles, and infrastructure needed for implementation work; De-implementation: Scaling back or discontinuing ineffective or low-value practices | None |
| **University of Ottawa** | MSc concentration, Implementation Science | <https://www.uottawa.ca/faculty-medicine/graduate-postdoctoral/programs-admission/program-information/epidemiology> | Canada | Graduate level (ie. MPH, MS, MSc, MD, PhD, DrPH, DSW, EdD, PsyD, JD) | Annually | $25,000 CAD | 1-10 | More than one year | Academia (ie. faculty, researchers, students, postdoctoral fellows) | Foundations of Implementation Science: Defining key terms, distinguishing implementation science from related fields, and explaining its purpose; Frameworks and Theories: Using models, frameworks, and planning tools to guide implementation and sustain change; Assessing Context and Readiness: Identifying what to implement and evaluating setting characteristics, capacity, and barriers; Stakeholder and Community Engagement: Involving partners, communities, and end users through participatory and collaborative approaches; Adaptation and Fidelity: Balancing fidelity to interventions while making appropriate adaptations; Implementation Strategies: Selecting, tailoring, and applying strategies to improve implementation and scale-up; Measurement and Outcomes: Measuring relevant outcomes and identifying gaps in existing measures; Study Design and Methods: Designing and analyzing implementation studies using mixed methods and systems thinking; Economic and Policy Considerations: Addressing costs, incentives, and policy influences on implementation; De-implementation: Scaling back or discontinuing ineffective or low-value practices | None |
| **University of Pennsylvania** | Implementation Science Institute (ISI) | <https://www.med.upenn.edu/pisce/implementation-science-institute.html> | Remote (online) | Undergraduate level (ie. BA, BS), Graduate level (ie. MPH, MS, MSc, MD, PhD, DrPH, DSW, EdD, PsyD, JD), Post-doctoral, Professional development workshop / training | Annually | $1,890 | 100+ | 1 - 7 days | Academia (ie. faculty, researchers, students, postdoctoral fellows), Education sector professionals, Healthcare providers, Human services professionals, Public health professionals | Foundations of Implementation Science: Defining key terms, distinguishing implementation science from related fields, and explaining its purpose; Frameworks and Theories: Using models, frameworks, and planning tools to guide implementation and sustain change; Stakeholder and Community Engagement: Involving partners, communities, and end users through participatory and collaborative approaches; Implementation Strategies: Selecting, tailoring, and applying strategies to improve implementation and scale-up; Measurement and Outcomes: Measuring relevant outcomes and identifying gaps in existing measures; Study Design and Methods: Designing and analyzing implementation studies using mixed methods and systems thinking; Economic and Policy Considerations: Addressing costs, incentives, and policy influences on implementation; Capacity Building and Team Composition: Building the skills, roles, and infrastructure needed for implementation work; De-implementation: Scaling back or discontinuing ineffective or low-value practices | Maternal health |
| **University of Pennsylvania** | Certificate in Implementation Science | <https://www.itmat.upenn.edu/itmat-ed/implementation-science-certificate-program.html> | United States, Remote (online) | Certificate program | Multiple admissions per year | $24,224 | 1-10 | More than one year | Academia (ie. faculty, researchers, students, postdoctoral fellows), Public health professionals | Foundations of Implementation Science: Defining key terms, distinguishing implementation science from related fields, and explaining its purpose; Frameworks and Theories: Using models, frameworks, and planning tools to guide implementation and sustain change; Assessing Context and Readiness: Identifying what to implement and evaluating setting characteristics, capacity, and barriers; Stakeholder and Community Engagement: Involving partners, communities, and end users through participatory and collaborative approaches; Adaptation and Fidelity: Balancing fidelity to interventions while making appropriate adaptations; Implementation Strategies: Selecting, tailoring, and applying strategies to improve implementation and scale-up; Study Design and Methods: Designing and analyzing implementation studies using mixed methods and systems thinking; De-implementation: Scaling back or discontinuing ineffective or low-value practices | None |
| **University of South Florida** | Institute for Translational Research Education in Adolescent Substance Use (ITRE) | <https://www.usf.edu/cbcs/cfs/itre/index.aspx> | United States | Undergraduate level (ie. BA, BS), Graduate level (ie. MPH, MS, MSc, MD, PhD, DrPH, DSW, EdD, PsyD, JD), Certificate program | Annually | Funded | 11-20 | More than one year | Academia (ie. faculty, researchers, students, postdoctoral fellows), Community organizations, Education sector professionals, Healthcare providers, Human services professionals, Public health professionals | Foundations of Implementation Science: Defining key terms, distinguishing implementation science from related fields, and explaining its purpose; Frameworks and Theories: Using models, frameworks, and planning tools to guide implementation and sustain change; Assessing Context and Readiness: Identifying what to implement and evaluating setting characteristics, capacity, and barriers; Stakeholder and Community Engagement: Involving partners, communities, and end users through participatory and collaborative approaches; Adaptation and Fidelity: Balancing fidelity to interventions while making appropriate adaptations; Implementation Strategies: Selecting, tailoring, and applying strategies to improve implementation and scale-up; Study Design and Methods: Designing and analyzing implementation studies using mixed methods and systems thinking; Capacity Building and Team Composition: Building the skills, roles, and infrastructure needed for implementation work | Behavioral health, substance use |
| **University of Texas Health Science Center at Houston** | Certificate in Dissemination and Implementation Science | <https://catalog.uth.edu/public-health/programs/graduate-certificates/dissemination-implementation-science/> | United States | Graduate level (ie. MPH, MS, MSc, MD, PhD, DrPH, DSW, EdD, PsyD, JD), Certificate program | Rolling enrollment | $3,720 (resident)  $9,795 (non-resident) | 1-10 | More than one year | Academia (ie. faculty, researchers, students, postdoctoral fellows), Public health professionals | Foundations of Implementation Science: Defining key terms, distinguishing implementation science from related fields, and explaining its purpose; Frameworks and Theories: Using models, frameworks, and planning tools to guide implementation and sustain change; Assessing Context and Readiness: Identifying what to implement and evaluating setting characteristics, capacity, and barriers; Stakeholder and Community Engagement: Involving partners, communities, and end users through participatory and collaborative approaches; Adaptation and Fidelity: Balancing fidelity to interventions while making appropriate adaptations; Implementation Strategies: Selecting, tailoring, and applying strategies to improve implementation and scale-up; Measurement and Outcomes: Measuring relevant outcomes and identifying gaps in existing measures; Study Design and Methods: Designing and analyzing implementation studies using mixed methods and systems thinking; Economic and Policy Considerations: Addressing costs, incentives, and policy influences on implementation; Capacity Building and Team Composition: Building the skills, roles, and infrastructure needed for implementation work; De-implementation: Scaling back or discontinuing ineffective or low-value practices | None |
| **University of Texas Health Science Center at Houston** | Implementation Science Workshop | <https://www.uth.edu/implementation-science/training/annual-workshop> | United States, Remote (online) | Professional development workshop / training | Annually | Funded | 11-20 | A few months | Academia (ie. faculty, researchers, students, postdoctoral fellows), Human services professionals, Public health professionals, Research institutes | Assessing Context and Readiness: Identifying what to implement and evaluating setting characteristics, capacity, and barriers; Stakeholder and Community Engagement: Involving partners, communities, and end users through participatory and collaborative approaches; Implementation Strategies: Selecting, tailoring, and applying strategies to improve implementation and scale-up; Measurement and Outcomes: Measuring relevant outcomes and identifying gaps in existing measures; Study Design and Methods: Designing and analyzing implementation studies using mixed methods and systems thinking | None |
| **University of Utah** | Graduate Certificate in Implementation Science | <https://ctsi.utah.edu/education/msci/graduate-certificates> | United States | Graduate level (ie. MPH, MS, MSc, MD, PhD, DrPH, DSW, EdD, PsyD, JD), Certificate program, Professional development workshop / training | Rolling enrollment | $8,914.83 (resident)  $23,731 (non-resident) | 1-10 | More than one year | Academia (ie. faculty, researchers, students, postdoctoral fellows) | Foundations of Implementation Science: Defining key terms, distinguishing implementation science from related fields, and explaining its purpose; Frameworks and Theories: Using models, frameworks, and planning tools to guide implementation and sustain change; Assessing Context and Readiness: Identifying what to implement and evaluating setting characteristics, capacity, and barriers; Stakeholder and Community Engagement: Involving partners, communities, and end users through participatory and collaborative approaches; Implementation Strategies: Selecting, tailoring, and applying strategies to improve implementation and scale-up; Measurement and Outcomes: Measuring relevant outcomes and identifying gaps in existing measures; Study Design and Methods: Designing and analyzing implementation studies using mixed methods and systems thinking; Capacity Building and Team Composition: Building the skills, roles, and infrastructure needed for implementation work; De-implementation: Scaling back or discontinuing ineffective or low-value practices | None |
| **University of Washington** | PhD in Global Health Metrics and Implementation Science | <https://globalhealth.washington.edu/academic-programs/phd-global-health-metrics-and-implementation-science> | United States | Graduate level (ie. MPH, MS, MSc, MD, PhD, DrPH, DSW, EdD, PsyD, JD) | Annually | Funded | 1-10 | More than one year | Academia (ie. faculty, researchers, students, postdoctoral fellows), Funding agencies / Government institutions, Policy makers, Public health professionals, Research institutes | Foundations of Implementation Science: Defining key terms, distinguishing implementation science from related fields, and explaining its purpose; Frameworks and Theories: Using models, frameworks, and planning tools to guide implementation and sustain change; Assessing Context and Readiness: Identifying what to implement and evaluating setting characteristics, capacity, and barriers; Stakeholder and Community Engagement: Involving partners, communities, and end users through participatory and collaborative approaches; Adaptation and Fidelity: Balancing fidelity to interventions while making appropriate adaptations; Implementation Strategies: Selecting, tailoring, and applying strategies to improve implementation and scale-up; Measurement and Outcomes: Measuring relevant outcomes and identifying gaps in existing measures; Study Design and Methods: Designing and analyzing implementation studies using mixed methods and systems thinking; Economic and Policy Considerations: Addressing costs, incentives, and policy influences on implementation | None |
| **University of Washington** | The Implementation Science in Global Health Summer Institute | <https://depts.washington.edu/impsci/> | United States | Professional development workshop / training | Annually | Funded | 51-100 | 1 - 7 days | Academia (ie. faculty, researchers, students, postdoctoral fellows), Community organizations, Funding agencies / Government institutions, Healthcare institutions, Policy makers, Public health professionals, Research institutes | Foundations of Implementation Science: Defining key terms, distinguishing implementation science from related fields, and explaining its purpose; Frameworks and Theories: Using models, frameworks, and planning tools to guide implementation and sustain change; Stakeholder and Community Engagement: Involving partners, communities, and end users through participatory and collaborative approaches; Adaptation and Fidelity: Balancing fidelity to interventions while making appropriate adaptations; Implementation Strategies: Selecting, tailoring, and applying strategies to improve implementation and scale-up; Measurement and Outcomes: Measuring relevant outcomes and identifying gaps in existing measures; Study Design and Methods: Designing and analyzing implementation studies using mixed methods and systems thinking; Economic and Policy Considerations: Addressing costs, incentives, and policy influences on implementation | None |
| **University of Wisconsin–Madison** | Implementation Science & Community Health Outcomes Certificate | <https://ictr.wisc.edu/program/implementation-science-community-health-outcomes-certificate/> | United States | Graduate level (ie. MPH, MS, MSc, MD, PhD, DrPH, DSW, EdD, PsyD, JD), Certificate program, Professional development workshop / training | Rolling enrollment | $11,470 (resident)  $16,302 (non-resident) | 11-20 | Self-paced | Academia (ie. faculty, researchers, students, postdoctoral fellows) | Foundations of Implementation Science: Defining key terms, distinguishing implementation science from related fields, and explaining its purpose; Stakeholder and Community Engagement: Involving partners, communities, and end users through participatory and collaborative approaches; Implementation Strategies: Selecting, tailoring, and applying strategies to improve implementation and scale-up; Study Design and Methods: Designing and analyzing implementation studies using mixed methods and systems thinking | None |
| **Washington University in St. Louis** | PhD concentration, Dissemination and Implementation Science | <https://brownschool.washu.edu/academics/dissemination-and-implementation-science-phd-concentration/> | United States | Graduate level (ie. MPH, MS, MSc, MD, PhD, DrPH, DSW, EdD, PsyD, JD) | Annually | Funded | 1-10 | More than one year | Academia (ie. faculty, researchers, students, postdoctoral fellows) | Foundations of Implementation Science: Defining key terms, distinguishing implementation science from related fields, and explaining its purpose; Frameworks and Theories: Using models, frameworks, and planning tools to guide implementation and sustain change; Assessing Context and Readiness: Identifying what to implement and evaluating setting characteristics, capacity, and barriers; Stakeholder and Community Engagement: Involving partners, communities, and end users through participatory and collaborative approaches; Adaptation and Fidelity: Balancing fidelity to interventions while making appropriate adaptations; Implementation Strategies: Selecting, tailoring, and applying strategies to improve implementation and scale-up; Measurement and Outcomes: Measuring relevant outcomes and identifying gaps in existing measures; Study Design and Methods: Designing and analyzing implementation studies using mixed methods and systems thinking; Economic and Policy Considerations: Addressing costs, incentives, and policy influences on implementation; Capacity Building and Team Composition: Building the skills, roles, and infrastructure needed for implementation work; De-implementation: Scaling back or discontinuing ineffective or low-value practices | None |
| **Washington University in St. Louis** | T32 Center for Mental Health Services Research Fellowship | <https://brownschool.washu.edu/academics/phd-in-social-work/pre-and-postdoctoral-training/> | United States | Graduate level (ie. MPH, MS, MSc, MD, PhD, DrPH, DSW, EdD, PsyD, JD), Post-doctoral | Multiple admissions per year | Funded | 1-10 | More than one year | Academia (ie. faculty, researchers, students, postdoctoral fellows) | Foundations of Implementation Science: Defining key terms, distinguishing implementation science from related fields, and explaining its purpose; Frameworks and Theories: Using models, frameworks, and planning tools to guide implementation and sustain change; Assessing Context and Readiness: Identifying what to implement and evaluating setting characteristics, capacity, and barriers; Stakeholder and Community Engagement: Involving partners, communities, and end users through participatory and collaborative approaches; Adaptation and Fidelity: Balancing fidelity to interventions while making appropriate adaptations; Implementation Strategies: Selecting, tailoring, and applying strategies to improve implementation and scale-up; Measurement and Outcomes: Measuring relevant outcomes and identifying gaps in existing measures; Study Design and Methods: Designing and analyzing implementation studies using mixed methods and systems thinking; Economic and Policy Considerations: Addressing costs, incentives, and policy influences on implementation; Capacity Building and Team Composition: Building the skills, roles, and infrastructure needed for implementation work; De-implementation: Scaling back or discontinuing ineffective or low-value practices | Mental health |
| **Washington University in St. Louis** | Implementation Research Institute (IRI) | [https://iri.wustl.edu](https://iri.wustl.edu/) | United States | Fellowship | Annually | Funded | 1-10 | More than one year | Academia (ie. faculty, researchers, students, postdoctoral fellows) | Foundations of Implementation Science: Defining key terms, distinguishing implementation science from related fields, and explaining its purpose; Frameworks and Theories: Using models, frameworks, and planning tools to guide implementation and sustain change; Assessing Context and Readiness: Identifying what to implement and evaluating setting characteristics, capacity, and barriers; Stakeholder and Community Engagement: Involving partners, communities, and end users through participatory and collaborative approaches; Adaptation and Fidelity: Balancing fidelity to interventions while making appropriate adaptations; Implementation Strategies: Selecting, tailoring, and applying strategies to improve implementation and scale-up; Measurement and Outcomes: Measuring relevant outcomes and identifying gaps in existing measures; Study Design and Methods: Designing and analyzing implementation studies using mixed methods and systems thinking; Economic and Policy Considerations: Addressing costs, incentives, and policy influences on implementation; Capacity Building and Team Composition: Building the skills, roles, and infrastructure needed for implementation work; De-implementation: Scaling back or discontinuing ineffective or low-value practices | Mental health, substance use |
| **Washington University in St. Louis** | Dissemination and Implementation Science Training Institute | <https://sites.wustl.edu/disi/> | United States | Graduate level (ie. MPH, MS, MSc, MD, PhD, DrPH, DSW, EdD, PsyD, JD), Post-doctoral, Professional development workshop / training | Annually | $3,250 | 21-50 | 1 - 7 days | Academia (ie. faculty, researchers, students, postdoctoral fellows) | Foundations of Implementation Science: Defining key terms, distinguishing implementation science from related fields, and explaining its purpose; Frameworks and Theories: Using models, frameworks, and planning tools to guide implementation and sustain change; Assessing Context and Readiness: Identifying what to implement and evaluating setting characteristics, capacity, and barriers; Stakeholder and Community Engagement: Involving partners, communities, and end users through participatory and collaborative approaches; Adaptation and Fidelity: Balancing fidelity to interventions while making appropriate adaptations; Implementation Strategies: Selecting, tailoring, and applying strategies to improve implementation and scale-up; Measurement and Outcomes: Measuring relevant outcomes and identifying gaps in existing measures; Study Design and Methods: Designing and analyzing implementation studies using mixed methods and systems thinking; Economic and Policy Considerations: Addressing costs, incentives, and policy influences on implementation; Capacity Building and Team Composition: Building the skills, roles, and infrastructure needed for implementation work; De-implementation: Scaling back or discontinuing ineffective or low-value practices | None |
| **Washington University in St. Louis** | MSCI concentration, Implementation Science | <https://crtc.wustl.edu/degrees-certificates/msci/courses/> | United States | Graduate level (ie. MPH, MS, MSc, MD, PhD, DrPH, DSW, EdD, PsyD, JD), Post-doctoral | Annually | $49,005 | 1-10 | More than one year | Academia (ie. faculty, researchers, students, postdoctoral fellows) | Foundations of Implementation Science: Defining key terms, distinguishing implementation science from related fields, and explaining its purpose; Frameworks and Theories: Using models, frameworks, and planning tools to guide implementation and sustain change; Assessing Context and Readiness: Identifying what to implement and evaluating setting characteristics, capacity, and barriers; Stakeholder and Community Engagement: Involving partners, communities, and end users through participatory and collaborative approaches; Adaptation and Fidelity: Balancing fidelity to interventions while making appropriate adaptations; Implementation Strategies: Selecting, tailoring, and applying strategies to improve implementation and scale-up; Measurement and Outcomes: Measuring relevant outcomes and identifying gaps in existing measures; Study Design and Methods: Designing and analyzing implementation studies using mixed methods and systems thinking; Economic and Policy Considerations: Addressing costs, incentives, and policy influences on implementation; Capacity Building and Team Composition: Building the skills, roles, and infrastructure needed for implementation work; De-implementation: Scaling back or discontinuing ineffective or low-value practices | None |
| **Washington University in St. Louis** | HIV, Infectious Disease and Global Health Implementation Research Institute (HIGH IRI) | <https://sites.wustl.edu/highiri/> | United States | Post-doctoral | Annually | Funded | 11-20 | More than one year | Academia (ie. faculty, researchers, students, postdoctoral fellows), Policy makers, Public health professionals | Foundations of Implementation Science: Defining key terms, distinguishing implementation science from related fields, and explaining its purpose; Frameworks and Theories: Using models, frameworks, and planning tools to guide implementation and sustain change; Assessing Context and Readiness: Identifying what to implement and evaluating setting characteristics, capacity, and barriers; Stakeholder and Community Engagement: Involving partners, communities, and end users through participatory and collaborative approaches; Adaptation and Fidelity: Balancing fidelity to interventions while making appropriate adaptations; Implementation Strategies: Selecting, tailoring, and applying strategies to improve implementation and scale-up; Measurement and Outcomes: Measuring relevant outcomes and identifying gaps in existing measures; Study Design and Methods: Designing and analyzing implementation studies using mixed methods and systems thinking; Economic and Policy Considerations: Addressing costs, incentives, and policy influences on implementation; Capacity Building and Team Composition: Building the skills, roles, and infrastructure needed for implementation work; De-implementation: Scaling back or discontinuing ineffective or low-value practices | HIV, infectious disease, global health |
| **Yale School of Public Health** | MPH concentration, Implementation Science Track | <https://ysph.yale.edu/school-of-public-health/graduate-programs/master-of-public-health-mph-degree/implementation-science-track/> | United States | Graduate level (ie. MPH, MS, MSc, MD, PhD, DrPH, DSW, EdD, PsyD, JD) | Rolling enrollment | $110,640 | 11-20 | More than one year | Academia (ie. faculty, researchers, students, postdoctoral fellows) | Foundations of Implementation Science: Defining key terms, distinguishing implementation science from related fields, and explaining its purpose; Frameworks and Theories: Using models, frameworks, and planning tools to guide implementation and sustain change; Assessing Context and Readiness: Identifying what to implement and evaluating setting characteristics, capacity, and barriers; Stakeholder and Community Engagement: Involving partners, communities, and end users through participatory and collaborative approaches; Adaptation and Fidelity: Balancing fidelity to interventions while making appropriate adaptations; Implementation Strategies: Selecting, tailoring, and applying strategies to improve implementation and scale-up; Measurement and Outcomes: Measuring relevant outcomes and identifying gaps in existing measures; Study Design and Methods: Designing and analyzing implementation studies using mixed methods and systems thinking; Economic and Policy Considerations: Addressing costs, incentives, and policy influences on implementation; Capacity Building and Team Composition: Building the skills, roles, and infrastructure needed for implementation work; De-implementation: Scaling back or discontinuing ineffective or low-value practices | Public health |
| **Yale University** | MS concentration, Implementation and Prevention Science | <https://ysph.yale.edu/school-of-public-health/graduate-programs/master-of-science-in-public-health-msph/biostatistics/master-implementation-prevention-science-methods-pathway/> | United States | Graduate level (ie. MPH, MS, MSc, MD, PhD, DrPH, DSW, EdD, PsyD, JD), Post-doctoral, Webinar series | Monthly | $110,640 | 11-20 | More than one year | Academia (ie. faculty, researchers, students, postdoctoral fellows), Public health professionals | Foundations of Implementation Science: Defining key terms, distinguishing implementation science from related fields, and explaining its purpose; Frameworks and Theories: Using models, frameworks, and planning tools to guide implementation and sustain change; Assessing Context and Readiness: Identifying what to implement and evaluating setting characteristics, capacity, and barriers; Stakeholder and Community Engagement: Involving partners, communities, and end users through participatory and collaborative approaches; Adaptation and Fidelity: Balancing fidelity to interventions while making appropriate adaptations; Implementation Strategies: Selecting, tailoring, and applying strategies to improve implementation and scale-up; Measurement and Outcomes: Measuring relevant outcomes and identifying gaps in existing measures; Study Design and Methods: Designing and analyzing implementation studies using mixed methods and systems thinking; Economic and Policy Considerations: Addressing costs, incentives, and policy influences on implementation | Global health, mental health promotion, HIV prevention and care, cervical cancer prevention, cardriometabolic health |
